# Potent and Broad Inhibition of HIV-1 by a Peptide from the gp41 Heptad Repeat-2 Domain Conjugated to the CXCR4 Amino Terminus

**DOI:** 10.1371/journal.ppat.1005983

**Published:** 2016-11-17

**Authors:** George J. Leslie, Jianbin Wang, Max W. Richardson, Beth S. Haggarty, Kevin L. Hua, Jennifer Duong, Anthony J. Secreto, Andrea P. O. Jordon, Josephine Romano, Kritika E. Kumar, Joshua J. DeClercq, Philip D. Gregory, Carl H. June, Michael J. Root, James L. Riley, Michael C. Holmes, James A. Hoxie

**Affiliations:** 1 Department of Medicine, Perelman School of Medicine, University of Pennsylvania, Philadelphia, PA, United States of America; 2 Sangamo BioSciences Inc., Richmond, CA, United States of America; 3 Department of Microbiology, Perelman School of Medicine, University of Pennsylvania, Philadelphia, PA, United States of America; 4 Department of Pathology & Laboratory Medicine, Perelman School of Medicine, University of Pennsylvania, Philadelphia, PA, United States of America; 5 Department of Biochemistry and Molecular Biology, Kimmel Cancer Center, Thomas Jefferson University, Philadelphia, PA, United States of America; University of Zurich, SWITZERLAND

## Abstract

HIV-1 entry can be inhibited by soluble peptides from the gp41 heptad repeat-2 (HR2) domain that interfere with formation of the 6-helix bundle during fusion. Inhibition has also been seen when these peptides are conjugated to anchoring molecules and over-expressed on the cell surface. We hypothesized that potent anti-HIV activity could be achieved if a 34 amino acid peptide from HR2 (C34) were brought to the site of virus-cell interactions by conjugation to the amino termini of HIV-1 coreceptors CCR5 or CXCR4. C34-conjugated coreceptors were expressed on the surface of T cell lines and primary CD4 T cells, retained the ability to mediate chemotaxis in response to cognate chemokines, and were highly resistant to HIV-1 utilization for entry. Notably, C34-conjugated CCR5 and CXCR4 each exhibited potent and broad inhibition of HIV-1 isolates from diverse clades irrespective of tropism (i.e., each could inhibit R5, X4 and dual-tropic isolates). This inhibition was highly specific and dependent on positioning of the peptide, as HIV-1 infection was poorly inhibited when C34 was conjugated to the amino terminus of CD4. C34-conjugated coreceptors could also inhibit HIV-1 isolates that were resistant to the soluble HR2 peptide inhibitor, enfuvirtide. When introduced into primary cells, CD4 T cells expressing C34-conjugated coreceptors exhibited physiologic responses to T cell activation while inhibiting diverse HIV-1 isolates, and cells containing C34-conjugated CXCR4 expanded during HIV-1 infection *in vitro* and in a humanized mouse model. Notably, the C34-conjugated peptide exerted greater HIV-1 inhibition when conjugated to CXCR4 than to CCR5. Thus, antiviral effects of HR2 peptides can be specifically directed to the site of viral entry where they provide potent and broad inhibition of HIV-1. This approach to engineer HIV-1 resistance in functional CD4 T cells may provide a novel cell-based therapeutic for controlling HIV infection in humans.

## Author Summary

HIV-1 infection persists and requires life-long therapy. Approaches to prevent viral replication in the absence of treatment will likely require effective antiviral immune responses, but this goal has been confounded by HIV-1’s ability to target CD4 T cells that coordinate adaptive immunity. We describe a novel approach to confer HIV-resistance to CD4 T cells using peptides from the HIV-1 gp41 heptad repeat-2 (HR2) domain to inhibit infection. By linking a 34 amino acid HR2 peptide to the amino terminus of CCR5 or CXCR4 we were able to use the physiologic trafficking of these coreceptors to deliver the inhibitory peptide to sites of viral fusion where they exerted potent, specific and broad resistance irrespective of viral clade or tropism. This effect was highly dependent on the positioning of the peptide and most effective when conjugated to CXCR4. *In vitro* and in humanized mice, primary CD4 T cells were protected and expanded following HIV-1 infection. This work represents a proof of concept that T cells can be genetically engineered to resist infection *in vivo* and provides a rationale to explore this approach as a novel cell-based therapeutic in strategies to augment antiviral immune responses that target viral reservoirs and for long-term control of HIV-1.

## Introduction

HIV-1 infection persists in the face of suppressive anti-retroviral therapy, and following cessation of treatment, typically rebounds rapidly, generating new rounds of infection [1–4]. Viral persistence results from long-lived reservoirs that include memory CD4 T cells [5–7] and perhaps other cell types [8] that are established early after infection in humans [9] and, in pathogenic models of SIV infection in nonhuman primates [10]. While there is a single example of an individual cured of HIV infection following a stem cell transplant from a donor lacking CCR5 [11], *in vitro* [12, 13] and *in vivo* models [14] have strongly suggested that host immune responses will be required to eliminate or control virus in these sites. However, confounding immunologic approaches to control HIV-1 is the tropism of this virus, which targets CD4 T cells that are required to generate cellular and humoral anti-viral immune responses [15, 16].

To protect and/or enhance host immune responses to HIV-1, many approaches have been developed based on engineering primary CD4 T cells to become resistant to HIV-1 infection. Findings from our group and others, have shown that gene therapy for HIV-1 is feasible and capable of generating modified CD4 and CD8 T cells that persist in HIV-infected subjects [17–20], traffic to mucosal compartments where HIV-1 infection is frequently initiated and sustained [21], and are capable of exerting selection pressure on the virus [22]. Tebas and coworkers have recently shown that autologous peripheral CD4 T cells, rendered CCR5-negative through zinc-finger nuclease treatment and expanded *ex vivo*, could be re-infused safely into HIV-infected subjects where they persisted for months to years and expanded in the context of an interruption in anti-retroviral therapy [21]. In this study there was a striking correlation in frequency of disrupted CCR5 alleles and HIV-1 control after ART was removed, indicating that approaches to increase the number of protected CD4 T cells may lead to more durable control. However, while editing peripheral CD4 T cells to be CCR5-negative is feasible and can confer resistance to R5-tropic viruses, there are logistical concerns in that for maximal effect both alleles must be targeted and because this approach would be ineffective for X4- or dual-tropic HIV-1s [23]. Although complementary methods to ablate both CCR5 and CXCR4 are feasible, this dual mutagenesis occurs with relatively lower efficiency and carries the risk of causing possible functional defects [23, 24].

Many strategies to confer resistance to primary CD4 T cells involve stable expression of a transgene to produce an inhibitory protein or nucleic acid [25]. Building on the theme that soluble peptides from the HIV-1 envelope heptad repeat-2 domain (HR2) can inhibit viral entry by blocking formation of the gp41 6-helix bundle that is required for membrane fusion, inhibitory proteins containing the C-terminal 46 (C46) or 36 (C36) amino acids from HR2 conjugated to a membrane-associated scaffold protein have been shown to broadly inhibit R5- and X4-tropic HIV-1s [26–29]. These membrane anchored constructs exhibited antiviral effects when introduced into primary CD4 T cells [26, 27, 30, 31] and were well tolerated and non-immunogenic in a human trial [32]. However, their antiviral activity is dependent on high levels of expression on the cell surface, which can vary considerably in different cell types, and is further influenced by the design of the anchoring protein and *cis*-acting regulatory elements in the vector [33, 34]. Work in this area has gone forward in nonhuman primate simian-human immunodeficiency virus (SHIV) models using hematopoetic stem cells transduced with a C46-containing protein where a survival advantage of transduced cells was shown along with a reduction in plasma viremia [35]. However, no therapeutic benefit or clear antiviral effect was observed in a human trial when peripheral CD4 T cells were transduced *ex vivo* with a similar vector and re-infused into patients, most likely due to insufficient levels of gene-protected T cells [32].

Considering these findings, we reasoned that the potency and effectiveness of cell-based HR2-peptide inhibition could be increased if this peptide were brought to the precise site of viral entry by conjugation to molecules directly involved with HIV-1 entry, rather than to artificial scaffold proteins expressed nonspecifically on the cell surface. We introduced a 34 amino acid peptide from HR2 (C34) onto the amino termini of either CD4 or coreceptors, CCR5 and CXCR4. Strikingly, C34-conjugated coreceptors exhibited potent HIV-1 inhibition, with the greatest effect observed for C34-conjugated CXCR4. Considerably less inhibition was observed when C34 was fused to CD4. C34-coreceptor inhibition was dependent on peptide sequence, occurred irrespective of viral tropism for CCR5 or CXCR4 and on multiple viral clades, and occurred for HIV-1 isolates that were resistant to the soluble HR2 peptide inhibitor, enfuvirtide. Primary CD4 T cells expressing C34-conjugated coreceptors, particularly C34-CXCR4, were resistant to HIV-1 *in vitro* and *in vivo* in NOD/SCID IL-2Rγnull (NSG) mice, as seen by expansion of these cells during HIV-1 infection. Collectively, these findings demonstrate that stable expression of C34-containing coreceptors on peripheral CD4 T cells can confer potent and broad resistance to HIV-1 and may provide a novel strategy to augment anti-viral immune responses that complement approaches to target or control HIV-1 reservoirs in infected individuals.

## Results

### Coreceptors containing a 34 amino acid peptide from HR2 are expressed, bind to gp41 HR1, and are non-permissive for HIV-1 entry

**Fig 1:**
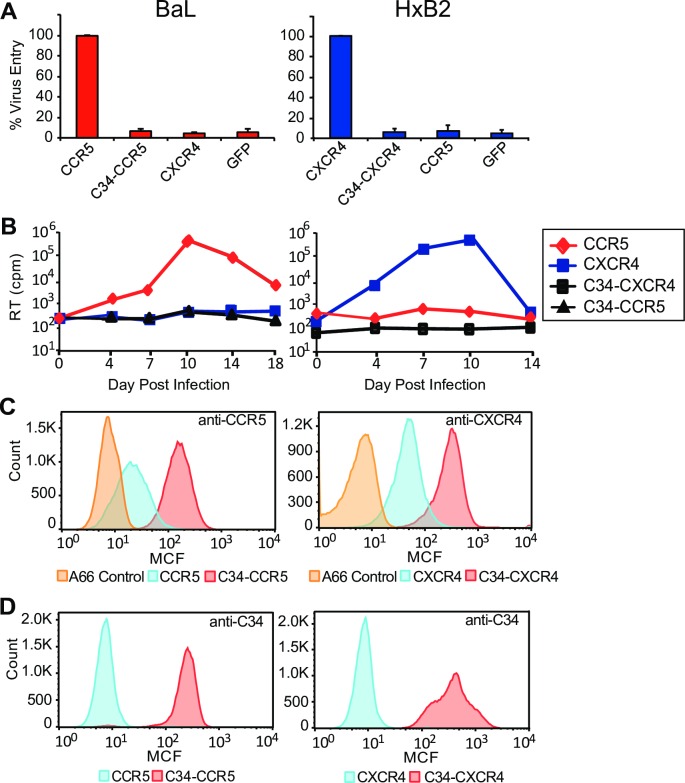
Inhibition of HIV-1 entry and infection by C34-conjugated coreceptors. **(A)** Entry of HIV-1 isolates BaL (R5-tropic) and HxB2 (X4-tropic) is shown on Cf2-Luc reporter cells transfected with CD4 and the indicated coreceptors or control (GFP). C34-conjugated CCR5 or CXCR4 do not permit entry. Error bars indicate standard error of the mean (S.E.M) and data shown are from 3 independent experiments. **(B)** Infection of CD4^+^ A66 cells stably expressing the indicated coreceptors is shown following inoculation by BaL or HxB2. (RT, reverse transcriptase activity). A representative experiment of 2 independent experiments performed is shown. **(C)** Surface expression of C34-conjugated and unconjugated CCR5 and CXCR4 is shown on A66-derived cell lines by FACS using an anti-CCR5 (2D7) or anti-CXCR4 (12G5) antibody. (D) Cells shown in **Panel C** expressing C34-conjugated or unconjugated coreceptors are shown stained with a monoclonal antibody to the C34 peptide. The specificity of this antibody is evident, since no overlap staining with this antibody is seen on A66-derived cell lines expressing unconjugated coreceptors.

A 34 amino acid peptide from the gp41 HR2 domain, corresponding to amino acids 628–661 in HxB2, was fused directly to the amino terminus of either CCR5 or CXCR4 flanked by an N-terminal alanine and C-terminal leucine, lysine linkers **(S1 Fig)**. When these C34-CCR5 or C34-CXCR4 coreceptors were transiently expressed with human CD4 on Cf2-Luc reporter cells and viral entry assessed using R5-tropic BaL or X4-tropic HxB2 HIV-1, respectively, only background levels of entry were detectable relative to unconjugated coreceptors (**Fig 1A**). When stably introduced via a lentiviral vector into human CD4^+^ SupT1 cells, from which endogenous CXCR4 had been ablated, (a line termed, A66 [36]) C34-CCR5 and C34-CXCR4 were expressed on the cell surface, as detected by a monoclonal antibody (mAb) specific for the C34 peptide and with mAbs reactive with the CCR5 or CXCR4 extracellular loops (**Fig 1C and 1D**). A66 cells bearing C34-conjugagted CCR5 or CXCR4 migrated in a chemotaxis assay in response to CCL4 and CXCL12, respectively, indicating that coreceptors containing this amino terminal peptide continued to exhibit physiologic responses to their cognate chemokines (**S2 Fig**). Consistent with the results of viral entry assays on Cf2-Luc cells, both C34-CCR5 and C34-CXCR4 were highly resistant to BaL and HxB2 infection (**Fig 1B**) indicating that the amino terminal HR2 peptide was likely able to access and inhibit HR1 domains of the pre-hairpin intermediate to block 6 helix-bundle formation and viral entry. Similar results were seen with additional R5- (YU2, JRFL) and dual-tropic (R3A) HIV-1 isolates (**S3 Fig**).

The ability of C34-conjugated coreceptors to interact with HR1 domains was also assessed quantitatively using 5-Helix, a partial mimetic of the gp41 6-helix bundle. 5-Helix is composed of three gp41 HR1 segments and two gp41 HR2 segments, and contains a single HR2 binding site of high specificity and affinity [37–39]. Using an interaction assay employing a rhodamine-conjugated 5-Helix construct, A66 cells stably expressing C34-CCR5 or C34-CXCR4 were shown to have approximately 52,000 and 55,000 molecules per cell, respectively, with K_D_ values less than 15 pM. Negligible binding was seen on A66 cells expressing unconjugated receptors (**S4 Fig**). Collectively, these findings indicate that C34-conjugated coreceptors could be processed and presented on the cell surface, retained the ability to interact with high affinity to HR1 domains that contribute to 6-helix bundle formation during fusion, and when positioned on the coreceptor amino terminus were able to prevent HIV-1 entry and infection.

### *Trans*-dominant homologous and heterologous inhibition of HIV-1 by C34-coreceptors

**Fig 2:**
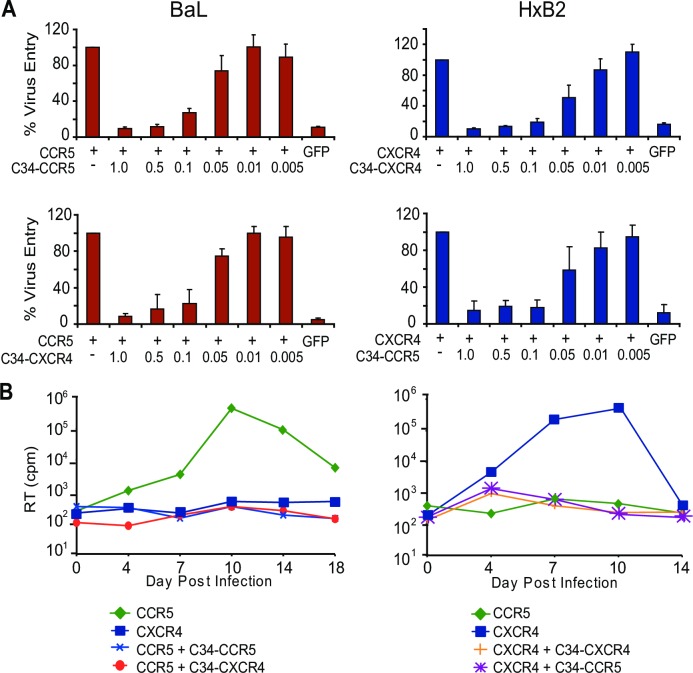
*Trans*-dominant homologous and heterologous inhibition of HIV-1 isolates by C34-conjugated coreceptors. **(A)** C34-conjugated CCR5 or CXCR4 plasmids were transfected at the indicated ratios with unlabeled coreceptors in transfected CF2-luc cells and inoculated with either HIV-1 BaL (**Left Upper** and **Lower Panels**) or HxB2 (**Right Upper** and **Lower Panels**). Both homologous (**Upper Panels**) and heterologous (**Lower Panels**) inhibition was observed up to a ratio of 1:10 of transfected plasmids for C34-conjugated to unconjugated coreceptors (see Methods Section for details). Error bars indicate S.E.M., and data shown is from 3 independent experiments. **(B)** A66 cells stably expressing the indicated C34-conjugated and/or unconjugated coreceptors were inoculated with HIV-1 BaL (**Left Panel**) or HxB2 (**Right Panel**) and infection monitored by RT activity. A representative experiment of 2 independent experiments performed is shown.

We next determined if C34-conjugated coreceptors could inhibit HIV-1 when co-expressed with unconjugated coreceptors, and if so, whether inhibition would occur in a homologous (i.e., C34-CXCR4 inhibiting X4-tropic HIV-1 from using unconjugated CXCR4; C34-CCR5 inhibiting R5-tropic HIV-1 from using unconjugated CCR5) or a heterologous manner (i.e., C34-CCR5 inhibiting X4-tropic HIV-1; C34-CXCR4 inhibiting R5-tropic HIV-1). Using the Cf2-Luc transfection assay, we assessed HIV-1 entry when increasing amounts of C34-conjugated coreceptors were cotransfected with a fixed amount of unconjugated coreceptors. Cf2-Luc cells were transfected with CD4 and unconjugated CCR5 or CXCR4 alone or in combination with varying ratios of C34-conjugated CCR5 or CXCR4, inoculated with R5-tropic (BaL) or X4-tropic (HxB2) HIV-1s, and RLUs quantified as an indicator of entry. As shown (**Fig 2A**, **Top Panels**), homologous inhibition by C34-coreceptors was evident for both R5- and X4-tropic HIV-1. Relative to fusion with unconjugated CCR5 or CXCR4, for both BaL and HxB2, respectively, levels of entry were comparable to cells transfected with only a GFP control, and inhibition was seen up to a 1:10 ratio of plasmids encoding C34-conjugated to unconjugated coreceptors. At a ratio of 1:10, subtracting background, BaL inhibition by C34-CCR5 was approximately 85% and HxB2 inhibition by C34-CXCR4 was >95%. Similarly, heterologous inhibition was also seen (**Fig 2A**, **Bottom Panels**). At input ratios up to 1:10 of C34-conjugated to unconjugated coreceptors, C34-CXCR4 could inhibit BaL from using CCR5 (85%); while C34-CCR5 could inhibit HxB2 from using CXCR4 (>95%). Both homologous and heterologous inhibition were progressively lost at higher dilutions of C34-conjugated receptors.

To evaluate homologous and heterologous inhibition of a spreading HIV-1 infection by C34-conjugated coreceptors, A66 cells were stably transduced to express CCR5 or CXCR4 alone or with C34-CXCR4 or C34-CCR5. C34-conjugagted coreceptors were clearly detectable, as determined by staining with an anti-C34 antibody (**S5 Fig**). As expected, BaL could infect A66 cells expressing CCR5 but not CXCR4; HxB2 could infect A66 cells expressing CXCR4 but not CCR5. However, co-expression of either C34-CCR5 or C34-CXCR4 with unconjugated coreceptors potently inhibited infection by either virus (**Fig 2B**).

Thus, inhibition of HIV-1 by C34-conjugated coreceptors could be mediated in a *trans*-dominant manner, irrespective of viral tropism, and was highly potent, with inhibition occurring at input ratios of expression plasmids of ≥1 to 10 C34-conjugated to unconjugated receptors. Of note, no inhibition occurred for SIVmac239 in SupT1 cells stably expressing C34-CCR5 (**S6 Fig**), consistent with the findings that peptides from HR2, including enfuvirtide, are poorly inhibitory for SIVmac [40, 41].

### C34-coreceptor inhibition of HIV-1 is dependent on the sequence and location of the HR2 peptide

**Fig 3:**
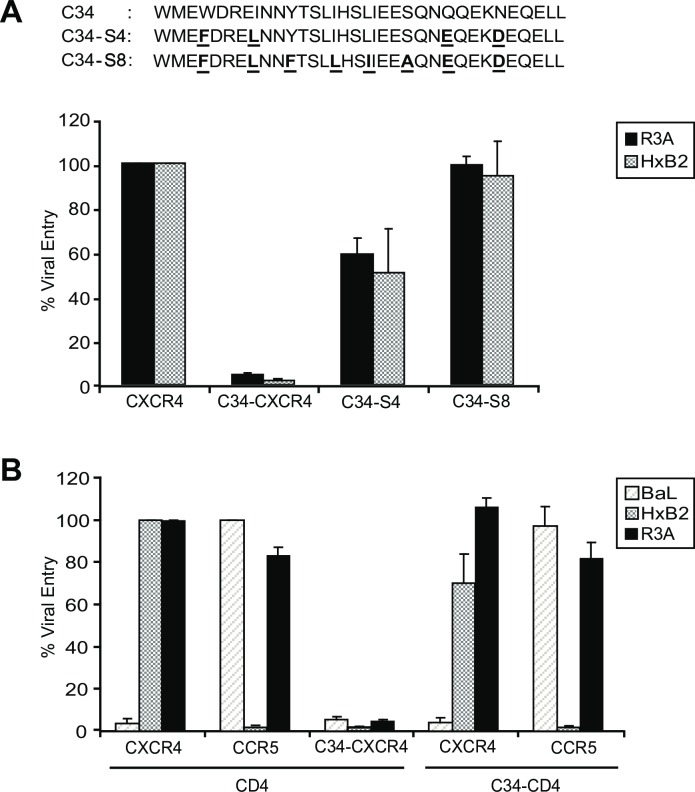
Effects of C34 peptide sequence and positioning on HIV-1 inhibition. **(A) Top Panel** shows the amino acid sequence of the C34 peptide and two derivatives containing 4 (C34-S4) or 8 (C34-S8) changes indicated that were conjugated to the CXCR4 amino terminus and coexpressed with CD4 in Cf2-Luc reporter cells. **Bottom Panel** shows entry relative to the unconjugated CXCR4 construct. A loss of inhibitory function is seen for both constructs with greater inhibition seen for C34-S8. **(B)** CD4 containing C34 conjugated to its amino terminus (C34-CD4) was coexpressed with CXCR4 or CCR5; unconjugated CD4 was coexpressed with CXCR4, CCR5 or C34-conjugated CXCR4. Entry inhibition was determined for R5- (BaL), X4- (HxB2), or dual-tropic (R3A) HIV-1s. Data are expressed as a percentage of entry for each virus on the indicated unconjugated coreceptors. For each Panel, error bars indicate S.E.M. from 3 independent experiments.

The specificity of C34-conjugated coreceptor inhibition of HIV-1 entry and infection was assessed by creating C34-CXCR4 constructs in which the sequence of the C34 peptide was altered at 4 (C34-S4) or 8 (C34-S8) positions shown previously to be critical for inhibiting 6-helix bundle formation and fusion [42, 43] (**Fig 3A, Top Panel**). These constructs were then transfected into Cf2-Luc cells with CD4 and evaluated for the ability to support infection by X4- (HxB2) or dual-tropic (R3A) HIV-1s. While C34-CXCR4 inhibited fusion for both viruses, fusion for CXCR4 conjugated to C34-S4 occurred at approximately 50% of unconjugated CXCR4, while CXCR4 conjugated to C34-S8 supported fusion at near wildtype levels (**Fig 3A, Bottom Panel**). Thus, residues within the C34 peptide required for blocking 6-helix bundle formation are also required for the inhibitory effect of the C34 peptide when conjugated to CXCR4. These results clearly indicate that C34-coreceptor inhibition is highly specific.

To evaluate the role of C34 peptide positioning in HIV-1 inhibition, we next determined whether this peptide would block HIV-1 entry when conjugated to the amino terminus of CD4. C34, followed by a Glu-Phe C-terminal linker, was inserted between amino acids 5 and 6 of the mature CD4 amino terminus (i.e., including the signal peptide and after the CD4 sequence, KKVVL), and this construct (designated C34-CD4) or wildtype CD4 were transiently expressed with CXCR4 or CCR5 in Cf2-Luc cells. Infection was assessed for R5- (BaL), X4- (HxB2), or dual-tropic (R3A) HIV-1s. C34-CD4 expression was verified with an anti-CD4 mAb reactive with the CD4 D1 domain and with an anti-C34 mAb (not shown). Relative to wildtype CD4, C34-conjugated CD4 when co-expressed with CXCR4 permitted fusion of HxB2 and R3A, and when co-expressed with CCR5, permitted fusion of BaL and R3A **(Fig 3B)**. In each case, fusion was 70–100% of the levels observed with unconjugated CD4. Thus, the ability of the C34 peptide to inhibit fusion was highly dependent on its positioning. These findings suggest that on the CD4 amino-terminus the C34 peptide was less able to access the HR1 binding site during 6-helix bundle formation and was therefore more limited in its ability to inhibit fusion, in contrast to when positioned on the more membrane-proximal coreceptor amino-terminus.

### HIV-1 Envs resistant to enfuvirtide remain susceptible to C34-coreceptor inhibition

**Fig 4:**
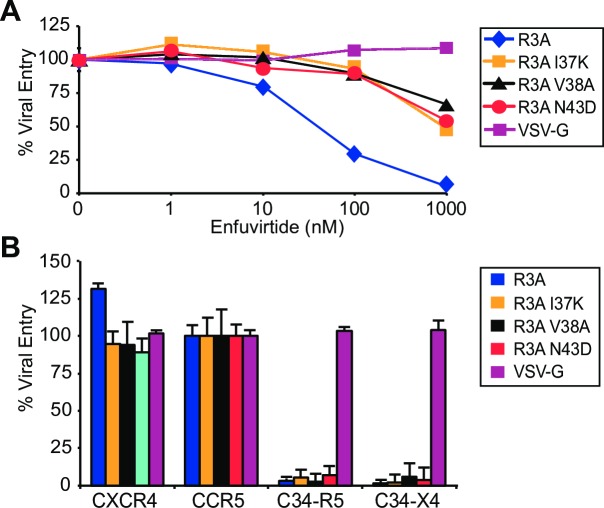
Sensitivity of enfuvirtide-resistant viruses to inhibition by C34-conjugated coreceptor. **(A)** Gp41 mutations known to confer HIV-1 resistance to enfuvirtide (I37K, V38A or N43D) were introduced into dual-tropic HIV-1 R3A and the effects on entry of pseudoviruses assessed on Cf2-Luc cells in the presence of the indicated concentrations of enfuvirtide. For each virus, RLUs are normalized to entry in the absence of enfuvirtide. Resistance conferred by these mutations is shown. A representative experiment from 2 independent experiments is performed. **(B)** Entry of pseudoviruses bearing these Envs was assessed on Cf2-Luc cells expressing the indicated coreceptors. All viruses were inhibited by C34-CCR5 and C34-CXCR4 constructs. In both panels VSV-G pseudoviruses served as a control. Error bars indicate S.E.M., and data shown are from 3 independent experiments.

Viral resistance to the soluble HR2-derived peptide enfuvirtide has been well documented *in vitro* and *in vivo* and typically involves mutations involving gp41 amino acids 26–45 in HR1 [29, 44]. To determine the extent to which mutations that confer resistance to a soluble HR2 peptide could also confer resistance to C34 peptide when conjugated to a coreceptor amino terminus, we introduced 3 mutations (I37K, V38A, and N43D) individually into the HIV-1 R3A HR1 domain. Mutations at each of these positions have been implicated in enfuvirtide resistance in patients undergoing anti-retroviral therapy [45–47]. As shown (**Fig 4A**), when assayed in a pseudovirus entry assay on transfected Cf2-Luc cells, each mutation conferred concentration-dependent resistance on CCR5 fusion by enfuvirtide (i.e., IC_50_ for parental R3A was 71 nM, but was ≥1000 nM for I37K, V38A and N43D Envs). However, when entry was assayed on C34-conjugated CCR5 or C34-conjugated CXCR4, all Envs were highly susceptible to inhibition **(Fig 4B**). As a control, no inhibition by enfuvirtide or C34-coreceptors was seen for a VSV-G Env. Thus, these findings strongly suggest that when anchored to a coreceptor amino terminus and in the context of a membrane-associated intermolecular interaction, an HR2-derived peptide exhibited enhanced potency that extended to viruses resistant to soluble HR2 peptides.

### C34-conjugated coreceptors exerted potent and broad inhibition of HIV-1 in primary CD4 T cells *in vitro*

**Fig 5:**
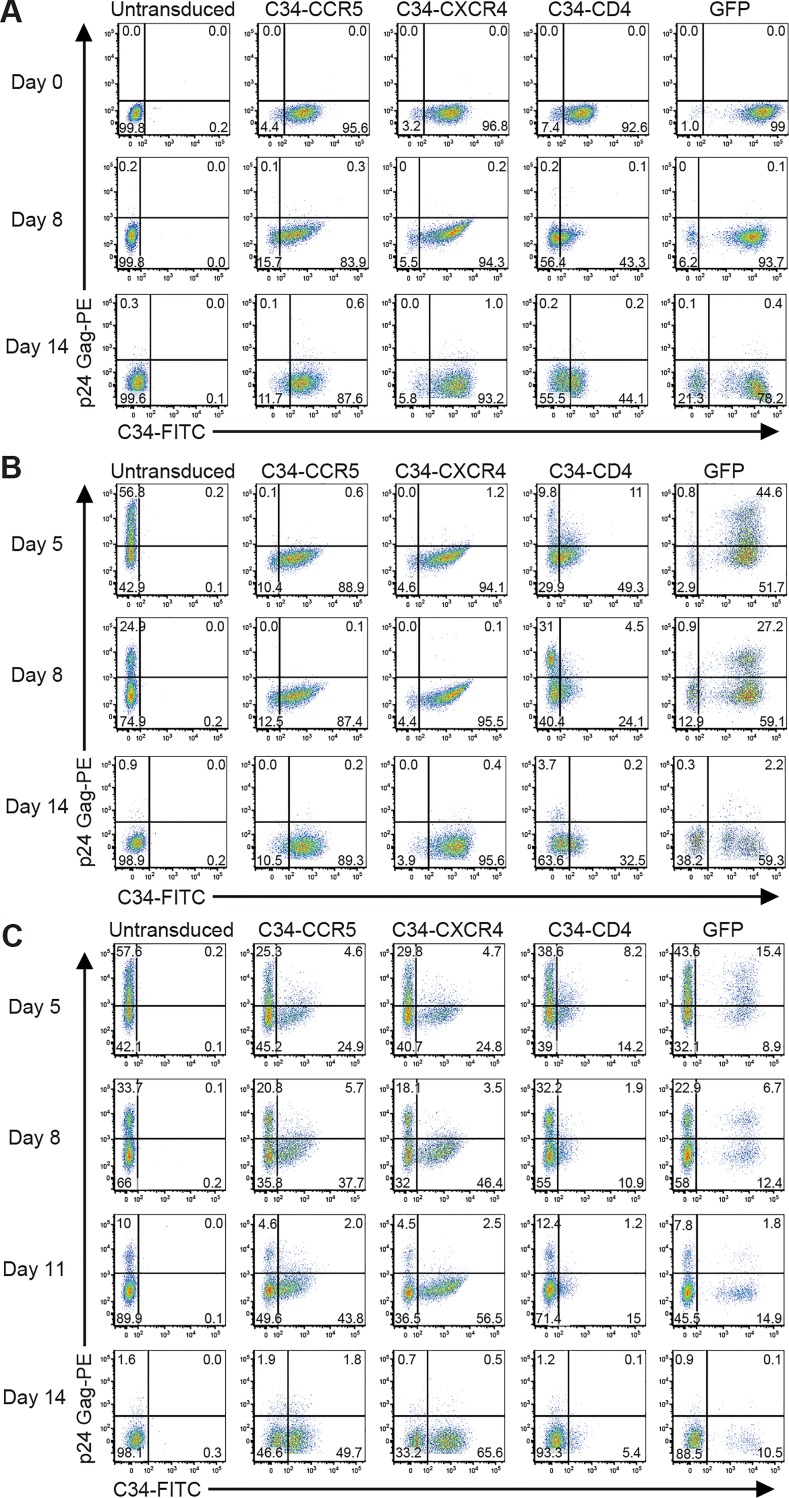
Expression of C34-conjugated coreceptors inhibits HIV-1 in primary CD4 T cells. Primary human CD4 T cells either untransduced or transduced with the indicated C34-conjugated constructs or GFP control, were either inoculated or not inoculated with HIV-1 JRFL, and monitored by flow cytometry for C34 peptide surface expression and intracellular HIV-1 p24-Gag. **(A)** Expression of C34-coreceptors or C34-CD4 in cells cultured in the absence of HIV-1 inoculation at Days 0, 5 and 14. **(B)** Cells inoculated with HIV-1 JRFL show stable expression of C34-constructs at Days 5 and 14 with marked inhibition of p24-Gag expression in C34-CCR5 and C34-CXCR4 transduced cultures relative to untransduced, GFP- or C34-CD4 transduced cells. **(C)** Cells transduced with C34-CCR5, C34-CXCR4, C34-CD4 or GFP were added at a 1:3 ratio to untransduced cells for a final percentage of 25% transduced cells and inoculated with JRFL. Expansion of cells expressing C34-CCR5 or C34-CXCR4 is shown over time. No expansion of the C34-CD4- or GFP-transduced cells was seen. A representative experiment from 2 independent experiments is shown, each with a different healthy donor.

Given the ability of C34-coreceptors to inhibit HIV-1 entry when expressed in Cf2-Luc and T cell lines, we evaluated their effects on primary CD4 T cells. Purified CD4 T cells from healthy donors, stimulated with anti-CD3/CD28 coated beads and maintained in IL2-containing media, were transduced with lentiviral vectors encoding C34-conjugated CCR5 or CXCR4 or, as controls, GFP or C34-conjugated CD4, given its poor ability to inhibit HIV-1 (**Fig 3**). Uninfected CD4 T cells transduced with C34-CCR5 or C34-CXCR4 maintained expression at levels 85–95% of total T cells for up to 14 days. In contrast, expression of C34-CD4 decreased over time; whereas 92.6% of cells were positive at day 0, only 44.1% of T cells expressed this construct at day 14 **(Fig 5A)**. Additionally, in response to CD3/CD28 stimulation, CD4 T cells transduced with C34-coreceptor proliferated and showed no differences in expression of intracellular cytokines (IL2, TNFα, IFNγ, and CCL4) upon T cell restimulation (**S7 Fig**), suggesting that this construct does not interfere with T cell activation or function.

Cells were inoculated with R5-, X4- or dual tropic HIV-1s from different clades and infection monitored for 14–17 days by flow cytometry for intracellular p24-Gag. Results for R5-tropic JRFL 5 days after viral inoculation are shown in **Fig 5B (Top Panels)**. For non-transduced, GFP-, or C34-CD4-transduced cells, 57.0%, 45.4% or 20.8% p24-Gag^+^ cells, respectively, were seen. In contrast, in C34-CCR5 or C34-CXCR4 transduced cells, p24-Gag expression was markedly reduced to 0.7% and 1.2% in C34-CCR5- or C34-CXCR4-transduced cells, respectively. Over time, this inhibition persisted with <1% p24-Gag^+^ cells at Days 8 and 14 (**Fig 5B, Middle and Lower Panels**).

The kinetics of JRFL p24-Gag^+^ expression and inhibition are shown in **S8 Fig**. While we observed modest protection of CD4 T cells expressing C34-CD4, much more robust resistance to HIV infection was seen in CD4 T cells expressing either C34-CCR5 or C34-CXCR4. The finding that JRFL infection was inhibited by both C34-CCR5 and C34-CXCR4 demonstrated that the *trans*-dominant homologous and heterologous inhibition of HIV-1 seen on T cell lines also occurred on primary cells. Similar results were seen for other X4-, R5- and dual-tropic HIV-1 isolates (**Table 1**).

**Table 1:** Inhibition of HIV-1 infection of primary CD4 T cells by C34-conjugated coreceptors*

Virus	DPI	Clade	Tropism	C34-CCR5	C34-CXCR4	GFP	Untransduced
**JRFL**	5	B	R5	0.7	1.3	45	**57**
**BaL**	5	B	R5	0.6	0.3	63	**69**
**US1**	8	B	R5	0.0	0.1	30	**36**
**CMU-02**	8	A/E	X4	0.3	0.1	34	**40**
**MN**	8	B	X4	0.2	0.1	21	**27**
**R3A**	8	B	R5/X4	0.2	0.2	18	**24**
**SF2**	8	B	R5/X4	0.1	0.1	50	**53**

* Purified CD4 T cells were inoculated with the indicated viruses and intracellular p24-Gag expression assessed. The percentage of positive cells are shown at the indicated day post infection (DPI). Controls are cells untransduced or transduced with GFP.

**Fig 6:**
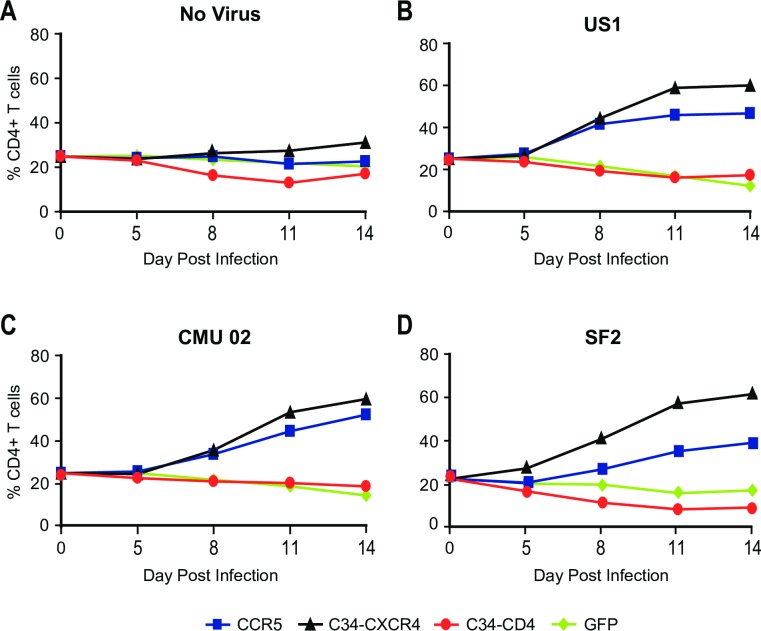
Selection of primary CD4 T cells expressing C34-conjugated coreceptors during HIV-1 infection. Primary human CD4 T cells transduced with the indicated C34-conjugated coreceptors, C34-conjugated CD4, or GFP as a control were mixed with untransduced cells for a final concentration of 25%. Cultures were inoculated with the indicated HIV-1 isolates and the proportion of C34-expressing cells determined over time by flow cytometry. Uninfected cultures are shown (**Panel A)** as are cultures infected by HIV-1 isolates US1 (**Panel B**), CMU-02 (**Panel C**) and SF2 (**Panel D**). In all HIV-1 infected cultures an expansion of C34-CCR5 and C34-CXCR4 cells was seen in contrast to cultures containing cells transduced with C34-CD4 or GFP. A representative experiment from 2 independent experiments is shown, each with a different healthy donor.

To determine whether C34-coreceptor expressing primary CD4 T cells were selectively enriched during HIV-1 infection, transduced and non-transduced cells were mixed prior to infection at a ratio of 1:3 and the proportion of C34-expressing cells was assessed over time in multiple donors. As shown (**Fig 5C**), following JRFL infection, C34-coreceptor transduced cells increased over time and by Day 14, C34-CCR5- and C34-CXCR4-expressing cells had increased to 51.5% and 66.1%, respectively. In contrast, C34-CD4-transduced cells decreased to 5.5%, with a similar decrease seen when cells were transduced with GFP (**Fig 5C, Lower Panels**). An expansion of C34-CCR5- or C34-CXCR4-expressing cells was also seen in cultures inoculated with additional primary R5- (US1), X4-tropic (CMU-02), and dual R5/X4-tropic (SF2) HIV-1 isolates **(Fig 6)**. We observed highly restricted replication of dual tropic HIV-1 R3A in cultures containing C34-coreceptor-transduced CD4 T cells, and the expansion of these cells when diluted with untransduced CD4 T cells are shown in **S9A** and **S9B Fig,** respectively. Of note, in assays performed using multiple donors and with different HIV-1 isolates, a greater expansion of cells expressing C34-CXCR4 was seen than for cells expressing C34-CCR5 **(S1 Table)**.

Thus, expression of C34-CCR5 or C34-CXCR4 in primary cells does not impair their ability to proliferate or to produce cytokines following T cell activation, and cells expressing these constructs exhibited resistance to multiple primary HIV-1 isolates irrespective of tropism, and showed selective expansion in the setting of HIV-1 infection with greater expansion seen for C34-CXCR4-transduced cells.

### CD4 T cells expressing C34-CXCR4 exhibited increased survival in HIV-1 infected NSG mice

**Fig 7:**
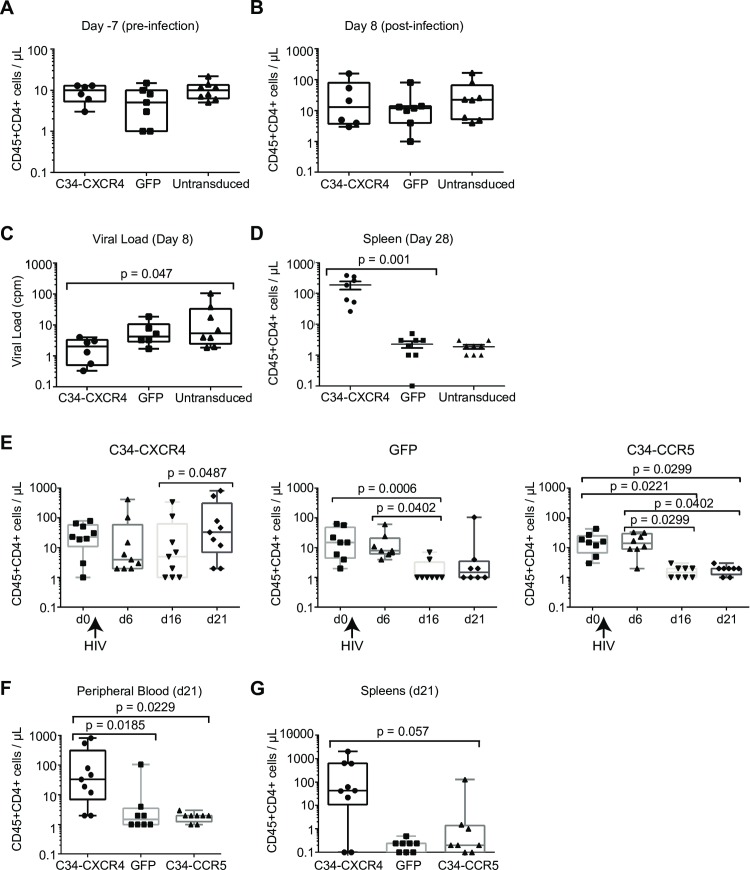
CD4 T cells expressing C34-CXCR4 are protected in HIV-1-infected NSG mice. **(Panels A-D).** NSG mice were infused with human CD4 T cells that were either untransduced or transduced with C34-CXCR4 or GFP. **(Panel A)** Engraftment was assessed after 30 days, which was 7 days prior to inoculation with a mix of HIV-1 isolates US1 (R5-tropic) and CMU-02 (X4-tropic). (**Panels B** and **C**) CD4 T cell numbers and plasma viral loads are shown 8 days after inoculation. **(Panel D)** CD4 T cells in spleen are shown at necropsy (Day 28). (**Panels E-F**) In a separate experiment, NSG mice were infused with CD4 T cells transduced with C34-CXCR4, C34-CCR5, or GFP and following engraftment inoculated with the same HIV-1 viral mix. (**Panel E**) Numbers of CD4 T cells are shown starting at Day 0, (2 days before inoculation), and at the indicated days after inoculation. (**Panels F** and **G**) CD4 T cells in blood and in spleen are shown on the day of necropsy (Day 21).

To evaluate the ability of C34-conjugated coreceptors to protect CD4 T cells *in vivo*, we employed the NSG mouse model that has been used extensively to evaluate the ability of a wide range of antiviral agents to protect T cells from HIV-1 infection *in vivo* [23, 48–51]. We infused 10 million untransduced, GFP-transduced, or C34-CXCR4 transduced T cells into cohorts of mice. Once T cell engraftment was confirmed and quantified (**Fig 7A**) mice were challenged with mixture of R5 (US1) and X4-tropic (CMU-02) HIV-1 isolates. After eight days viral load was measured, at which time CD4 T cell levels were comparable (**Fig 7B**). Mice engrafted with C34-CXCR4 expressing T cells had significantly lower viral loads than mice engrafted with either untransduced or GFP-transduced T cells (**Fig 7C**). After an additional 20 days, mice were sacrificed and the numbers of human CD4 T cells in spleens was measured (**Fig 7D**), given that in this model, spleens have been shown to contain the majority of engrafted T cells. Mice engrafted with C34-CXCR4 expressing T cells showed a marked increase in CD4 T cells compared to mice engrafted with untransduced or GFP-transduced T cells. Given that our *in vitro* data in peripheral blood CD4 T cells indicated that survival of cells expressing C34-CXCR4 was superior to cells expressing C34-CCR5 (**S1 Table**), we compared survival of C34-CXCR4 and C34-CCR5 expressing cells in a second experiment. After CD4 T cell engraftment and HIV-1 infection, mice were bled at 10-day intervals to assess T cell survival and expansion. Remarkably, CD4 T cells expressing C34-CCR5 survived poorly with levels at days 16 and 21 that were comparable to GFP-transduced cells (**Fig 7E)**. However, in marked contrast, C34-CXCR4-transduced cells persisted throughout the period of HIV-1 infection in both peripheral blood and spleens (**Fig 7E, 7F and 7G**). Together, these data demonstrate that T cells expressing C34-CXCR4 are highly resistant to HIV-1 infection *in vivo* and exhibit a survival advantage over C34-CCR5 expressing cells.

## Discussion

The persistence of HIV-1 in reservoirs remains the principal obstacle to curing infected individuals [52–55]. Although a substantial proportion of viruses archived in these compartments are defective and/or unable to replicate in CD4 T cells [56], a genetically diverse array of replication competent viruses persists for the lifetime of the infected host and is poised to replicate upon discontinuation of antiretroviral therapy [57, 58]. While pharmacologic approaches are being explored to reverse HIV-1 latency and drive reservoirs to a more active state that could be vulnerable to antiviral interventions [55, 59, 60], there has been no proof of concept to date that these agents alone can impact the size of the reservoir or its capacity to generate new infectious viruses. Indeed, *in vitro* studies have strongly suggested that whether the goal is elimination of HIV-1 reservoirs or their long-term control in the absence of antiretroviral therapy, an immunologic response will likely be required [12, 13] and will need to persist and be broad enough to recognize the genetic diversity within the reservoir. This response will also need to be resistant to the immunopathogenic effects of HIV infection on CD4 T cells that provide help to initiate and sustain adaptive immunity.

In this report we show that conjugating a fusion-inhibitory peptide from the gp41 HR2 domain to the amino terminus of HIV-1 coreceptors CCR5 or CXCR4 exerts potent, broad, and specific inhibition of genetically diverse HIV-1 isolates. The conjugated C34 peptide exhibited picomolar binding affinity to soluble 5-Helix, which presents HR1 domains in a trimeric context that likely reflects a structure formed as the gp41 pre-hairpin fusion intermediate transitions to the 6-helix bundle during viral entry [37–39]. C34-conjugated CCR5 and CXCR4 were unable to be used for infection by R5- and X4-tropic viruses, respectively, and exhibited potent inhibition over unconjugated receptors with inhibition at input ratios of expression plasmids as low as 1 C34-conjugated to 10 unconjugated receptors. Strikingly, this *trans*-dominant inhibition occurred irrespective of viral tropism and across genetically diverse clades. The potency of inhibition was further reflected by the ability of C34-conjugated CCR5 to inhibit viruses that were resistant to the soluble HR2 peptide, enfuvirtide, consistent with more effective binding of the conjugated peptide to gp41 in the setting of a highly localized intermolecular interaction. *In vitro* and in NOG/SCID mice, CD4 T cells stably expressing C34-conjugated coreceptors appeared to be resistant to HIV-1 infection by their selective outgrowth during HIV-1 infection. Collectively, these findings demonstrate a novel approach to enhance the fusion-inhibiting properties of HR2 peptides and to confer broad and durable protection from HIV-1 infection to CD4 T cells by directly targeting the peptides to the precise site of fusion and viral entry.

**Fig 8:**
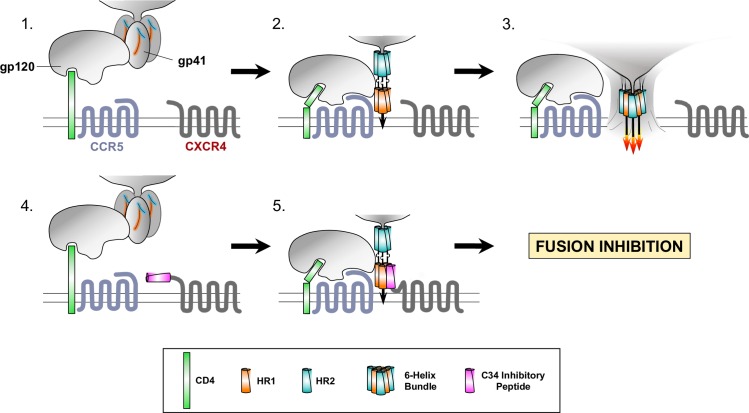
Model of HIV-1 fusion and entry inhibition by C34-containing coreceptors. **(Panels 1, 2 and 3)** Diagrams depicting stages of HIV-1 fusion are shown. **Panel 1**: HIV-1 gp120 engages CD4 through its CD4 binding site. Coreceptors CCR5 and CXCR4 colocalize with CD4 in lipid raft microdomains. Gp41, attached noncovalently to gp120, contains HR1 and HR2 regions, separated by steric constraints within the Env trimer. **Panel 2**: Conformational changes following CD4 binding enable coreceptor binding (CCR5 in the diagram) to occur through interactions of gp120 with CCR5 extracellular loops and its amino terminus resulting in formation of the gp41 pre-hairpin intermediate in which the N-terminal fusion peptide is inserted into target cell membrane and HR1 and HR2 are released. **Panel 3**: The 6-helix bundle forms by packing of individual HR2 peptides into outer grooves of the centrally-located HR1 trimers, providing energy to merge viral and cellular membranes and to create a fusion pore that initiates viral entry. **(Panels 4 and 5)** Fusion/entry inhibition by C34-containing coreceptors. **Panel 4**: A neighboring coreceptor (CXCR4 in the diagram) is shown containing a C34 peptide fused to its amino terminus. **Panel 5**: With the release of gp41 and formation of the prehairpin intermediate, the N-terminal C34 peptide is positioned to interact directly with binding sites within the HR1 trimer, thus preventing 6-helix bundle formation and inhibiting fusion.

How do C34-conjugated coreceptors inhibit HIV-1 irrespective of viral tropism and with such high stoichiometric potency? Several reports have shown that CD4, CCR5 and CXCR4 reside in cholesterol-rich microdomains, termed lipid rafts, on the plasma membrane [61–63]. Although some reports have indicated that in contrast to CCR5, CXCR4 may be only partially present in these domains [64–66], there is general agreement that in the context of HIV-1 gp120 and virion binding to CD4, lipid rafts can serve as sites for the recruitment, concentration and colocalization of CCR5 and CXCR4 to facilitate cooperative interactions with the envelope trimer that are required for entry [64, 66]. Indeed, disrupting coreceptor localization in lipid rafts by cholesterol depletion potently inhibits infection and entry of both R5- and X4-tropic HIV-1s [63, 64]. It is likely that C34-conjugated CCR5 and CXCR4 retain their physiologic trafficking and, as a result, are able to colocalize in lipid rafts with unconjugated receptors. In addition, HIV-1 entry requires highly cooperative interactions with multiple coreceptor molecules [67, 68] and there is evidence for direct interactions between CCR5 and CXCR4 [64] in lipid rafts including their ability to form heterodimers [69, 70]. Collectively these findings suggest that HR2 peptides conjugated to CCR5 or CXCR4 are well positioned to disrupt cooperative interactions between the viral envelope and coreceptors regardless of viral tropism. Lastly, it has been well documented that HR2 peptides target a transient intermediate state (i.e. the pre-hairpin fusion intermediate), and as a consequence, the potency of these inhibitors depends not only on how tightly they bind, but on how rapidly they associate [37–39] [71–73]. Thus, our findings are consistent with the model diagrammed in **Fig 8** that C34-conjugated coreceptors exploit physiologic trafficking of chemokine receptors to present HR2 peptides at the site of HIV-1 entry, and that the proximity of the C34-conjugated amino termini on CXCR4 or CCR5 is well positioned to interrupt formation of the 6-helix bundle and to take advantage of a kinetic window required for this structure to be generated.

To inhibit HIV-1 fusion, an HR2 peptide conjugated to a cell surface molecule must be able to be presented to trimeric heptad repeat-1 domains (HR1) on gp41 that are exposed during formation of the pre-hairpin fusion intermediate and prior to its conformational transition to the 6-helix bundle [74, 75]. Membrane-associated structures of CXCR4 [76, 77] and CCR5 [78] have been resolved at the atomic level for CCR5 in association with the small molecule inhibitor maraviroc [78] and for CXCR4 bound to a small molecule or a cyclic peptide antagonist [77] and the viral chemokine vMIP-II [76]. Although the amino termini of chemokine receptors play important roles in chemokine binding, a large number of N-terminal residues were missing in CXCR4 structures bound to vMIP-II and to US28 or CX3CL1 (i.e., 22 and 14 aminio acids, respectively), presumably due to its flexibility. Similarly, nearly the entire N-termini were also not resolved in the small molecule inhibitor-bound structures of CCR5 and CXCR4 (18 and 26 residues, respectively). Flexibility of the CXCR4 and CCR5 amino termini, which is a structural theme for many G-protein coupled receptors, likely plays a role in binding to gp120 by facilitating an interaction with the gp120 bridging sheet that forms following CD4 activation [79–81]. As noted above and diagrammed in **Fig 8**, this flexibility, as well as the membrane-proximal positioning of the CXCR4 and CCR5 amino termini, could serve to present the C34 peptide to HR1 domains following the insertion of the proximal amino terminal gp41 fusion peptide into the cell membrane. Although structural information on this pre-hairpin fusion intermediate is lacking, modeling strongly suggests that a spatial relationship between the C34 peptide on the coreceptor amino terminus and the trimeric HR1 domain anchored in the cell membrane is favorable for such an interaction to occur (personal communication, Irina Kufareva, UCSD, San Diego, CA). Interestingly, when the C34 peptide was conjugated to the amino terminus of CD4, it exhibited considerably less potency in preventing fusion, even though this construct was competent for binding to CD4-specific mAb and initiating fusion (**Fig 3**). It is likely that in this context the C34 peptide was poorly positioned on the more extended CD4 molecule and/or that gp120 binding itself prevented the peptide from accessing gp41 HR1 domains. Overall, our findings are consistent with the view that in the context of an intermolecular interaction between the envelope glycoprotein trimer, CD4 and coreceptors, that the positioning of the C34 peptide and its tethering to a flexible domain on the anchoring chemokine receptor was critical for inhibiting fusion.

HIV-1 peptides containing 36 or 46 amino acids from HR2 have been shown to inhibit HIV-1 infection in primary T cells when conjugated to membrane-associated scaffold proteins derived from the nerve growth factor receptor or CD34 [26, 27, 30, 33]. The effect of these constructs has been proposed to result from inhibition of 6-helix bundle formation and viral entry following the insertion of gp41 fusion peptides into the target cell membrane [25, 26, 29]. However, their antiviral activity has been shown to be dependent on high and stable levels of surface expression, which can vary considerably in different cell types [33, 34]. Although in human trials these constructs were well tolerated and non-immunogenic when transduced ex vivo in peripheral CD4 T cells and re-infused into patients, no selective advantage of transduced cells or antiviral effects were observed [32]. In nonhuman primates infected with a simian-human immunodeficiency virus (SHIV) bearing an HIV-1 envelope, stem cells transduced with a C46-containing construct showed a modest survival advantage, although no antiviral effects were seen [35]. While a head to head comparison of these constructs with the C34-conjugated coreceptors described in this report has not been conducted, the stoichiometric relationship of one C34-conjugated coreceptor to several unconjugated coreceptors suggests a highly efficient mechanism of fusion inhibition. Interestingly, in primary CD4 T cells *in vitro* and, particularly in humanized mice, the survival of CD4 T cells expressing C34-CXCR4 was greater than for C34-CCR5. Although there could be steric and/or structural attributes of the N-termini of these receptors that account for these differences, it is also possible that the level of C34-CXCR4 surface expression was greater over time in primary cells.

To be efficacious in HIV-infected humans, T cells expressing C34-conjugated coreceptors that have been rendered resistant to HIV-1 infection will also need to exhibit physiologic functions that permit their expansion and persistence and the ability to promote effective adaptive immune responses. Peripheral blood CD4 T cells expressing C34-conjugated CCR5 or CXCR4 proliferated and exhibited levels of cytokines when stimulated by CD3/CD28-mediated T cell receptor activation that were identical to untransduced T cells (**S7 Fig**). C34-conjugated coreceptors expressed on cell lines were also shown to mediate chemotaxis in response to their cognate chemokines, CCL4 for C34-CCR5 and CXCL12 for C34-CXCR4, although C34-CXCR4 appeared to be less efficient than wildtype CXCR4 (**S2 Fig**). It remains to be determined whether the ability to mediate chemotaxis would be advantageous to primary T cells *in vivo* or to what extent this function would affect the ability of these cells to provide help for adaptive immune responses. However, the potency of protection from HIV-1 infection conferred by these C34-conjugated coreceptors provides a rich opportunity for future studies to explore the full anti-viral potential of these cells. This question is of particular interest given possible antiviral effects observed during an interruption of anti-retroviral therapy in patients who received autologous CD4 T cells that had been rendered CCR5-negative and HIV-1 resistant (to R5-tropic isolates) by zinc-finger nuclease treatment [21].

In summary, these studies demonstrate a proof of concept that peptides from gp41 HR2 can be delivered to the precise sites of HIV-1 entry when conjugated to chemokine receptors CCR5 or CXCR4, with C34-conjugated CXCR4 conferring particularly potent, broad and durable protection from HIV-1 infection to primary CD4^+^ T cells *in vitro* and in humanized mice. The ability to exploit the physiologic trafficking of coreceptors that are essential for HIV-1 entry, and spatial relationships of viral and cellular molecules that interact during fusion provide a novel approach to generate HIV-1 resistance. Whether the creation of HIV-1 resistant CD4 T cells can generate stable and long lasting antiviral immune responses in infected patients remains to be determined; however, the feasibility of safely administering gene-modified peripheral T cells expanded *ex vivo*, has been well shown in patients with hematologic malignancies [82, 83] and in HIV-1 infection [21, 22, 84, 85].

## Materials and Methods

### Ethics Statement

All humanized mouse experiments were approved by the University of Pennsylvania’s Institutional Animal Care and Use Committee (Protocol 802717) and were carried out in accordance with recommendations in the Guide for the Care and Use of Laboratory Animals of the National Institutes of Health.

### Generation of C34-coreceptor and C34-CD4 expression constructs

The HIV gp41 HR2 domain (C34) peptide sequence was conjugated to the N-terminus of CCR5, CXCR4, or CD4 with a two amino acids spacer. To generate the pVAX-C34-CXCR4 expressing construct, two paired oligonucleotide (oligo) sequences (C34-left-F1/R1 and C34-right-F1/R1, refer to **S2 Table** for oligo sequences) were allowed to anneal to each other and then used as inserts to ligate into a NheI and AflII-digested pVAX-X4b construct, which is a pVAX plasmid (Life Technologies, Carlsbad, CA) with the CXCR4 isoform B cDNA cloned in. To generate the scrambled C34 with 4 mutated amino acids (SC34mut4 LLEQEDKEQENQAEEIISHLLSTFNNELRDFEMW), the two oligo pairs were replaced with SC34mut4-left-F1/R1 and SC34mut4-right-F1/R1. To generate the scramble C34 with 8 mutated amino acids (SC34mut8 LLEQEDKEQENQSEEILSHILSTYNNLERDFEMW), the two oligo pairs were replaced with SC34mut8-left-F1/R1and SC34mut8-right-F1/R1. To generate the pVAX-C34-CCR5 expressing construct, a pair of primers (R5 cDNA_F1/R1) were used to PCR amplify CCR5 cDNA (single exon) from genomic DNA (gDNA) of the Jurkat T cell line. The PCR product was digested with AflII and XhoI and ligated to pVAX-C34-CXCR4 digested with the same restriction enzymes. To generate the pVAX-C34-CD4 construct, the CD4 signal peptide portion was PCR amplified from a CD4 cDNA clone with a pair of primers (CD4sig_F1/R1) and then digested with SpeI and BsaI. The C34 portion was PCR amplified from the pVAX-C34-CXCR4 construct with a pair of primers (C34_F1/R1) and then digested with BsaI and EcoRI. The mature CD4 portion was PCR amplified from the CD4 cDNA clone with a pair of primers (CD4_cDNA_F1/R1) and then digested with EcoRI and Bgl2. The three parts described above were then ligated into pVAX plasmid digested with NheI and BglII.

To generate C34 constructs for lenti production, we first introduced NheI and XbaI restriction sites to flank the GFP ORF portion of the pCCLSIN.cPPT.hPGK.EGFP.wPRE construct [86] using a QuickChange Site-Directed Mutagenesis Kit (Agilent Technologies, Santa Clara, CA) to facilitate subsequent cloning steps. The resultant pCCLSIN.cPPT.hPGK.EGFP.wPRE-Nhe1Xba1 construct was digested with NheI and XbaI and ligated with inserts derived from C34-coreceptor constructs (pVAX-C34-CXCR4 or pVAX-C34-CCR5) digested with the same restriction enzymes to generate pCCLSIN.cPPT.hPGK.C34X4.wPRE or pCCLSIN.cPPT.hPGK.C34R5.wPRE. To generate pCCLSIN.cPPT.hPGK.C34CD4.wPRE, pVAX-C34-CD4 was digested with AseI and XbaI, blunted with DNA Polymerase I Klenow fragment, and then ligated to the digested and blunted pCCLSIN.cPPT.hPGK.EGFP.wPRE vector to replace the EGFP portion. The pTRPE lentivirus vector is previously described [50], and contains the EF1α promoter and cloning sites at 5’ (Nhe I site) and 3’ (Sal I site) ends. Both the C34-R5 and C34-X4 fragments were subcloned into pTRPE using 5’ Nhe1 and 3’ Sal1 sites.

The pCCLSIN or pTRPE constructs described above were used to produce lentivirus, pseudotyped with the VSV-G envelope, by transient co-transfection of four plasmids in 293T cells as described [87]. Expression of the transgene is driven by either the human phosphoglycerate kinase (PGK) promoter in pCCLSIN vector or EF1α in pTRPE vector.

### Cells

The 293T cells (Invitrogen/Thermo Fisher Scientific, Carlsbad, CA) for producing virus and the CF2-Luc cells (kindly gifted by Dr. Dana H. Gabuzda, Dana-Farber Cancer Institute, Boston, MA) for the entry assays were grown in Dulbecco's modified Eagle medium (DMEM) (high glucose) supplemented with 10% fetal bovine serum (FBS), 2 mM glutamine, and 2 mM penicillin/streptomycin. The human T-cell line SupT1 were obtained from the American Type Culture Collection (ATCC, Manassas, VA) and grown in Roswell Park Memorial Institute (RPMI) 1640 medium supplemented with 10% FBS, 2 mM glutamine, and 2 mM penicillin/streptomycin. The SupT1 derived A66 CXCR4 negative cell line, in which the endogenous CXCR4 alleles were both disrupted (CXCR4^-/-^), was created as previous described [36]. To generate A66-derived stable cell lines, A66 cells were transduced with lentivirus to express either wild-type (pTRPE constructs) or C34 fused receptors (pCCLSIN.cPPT.hPGK.wPRE or pTRPE constructs). Receptor-expressing cells were then enriched/selected by either cell sorting or single cell cloning (limited dilution and expansion).

### Generation of stable cell lines expressing C34-conjugated coreceptors

The lentiviral vector pTRPE with the EF1α promoter has cloning sites at 5’ (NheI site) and 3’ (SalI site). NheI and SalI digested C34-CCR5 and C34-CXCR4 fragments from pCCL.SIN.cPPT.hPGK.WPRE were subcloned into these sites to make the pTRPE constructs. These lentiviral vectors were then transfected in 293T cells in six-well plates using 3 μg vector, 1 μg gag, 1 μg pol, and 0.5 μg VSV-Env plasmids with 20 μl Lipofectamine 2000 (Invitrogen) for 1 h. After incubation at 37°C for 24 h. the cell-free supernatant was added to A66 cells and spun in six-well plates at 1,500 g for 1 h. This procedure was repeated a second time at 48 h post-transfection. Individual cells were then isolated through limiting dilution and when the cultures had expanded single clones were chosen based on their expression profile.

### CF2-luc infection assay

To determine which coreceptors were used by HIV-1 to enter cells CF2-Luc cells in six well plates were transfected for 1hr at 37oC with 2 μg per well of pcDNA3.1 containing CD4 and 6 μg per well of pcDNA3.1 containing coreceptors (C34-conjugated or unconjugated) and 25 μl Lipofectamine 2000 (Invitrogen). For experiments in which the ratio of C34-conjugated to unconjugated coreceptors was varied, the total amount of transfected DNA was maintained at 6 μg per well with parental pcDNA3.1 plasmid added, as needed, to maintain the same total input of DNA (e.g., for a ratio of 1.0 of C34-conjugated to unconjugated coreceptor, 3 μg of each coreceptor-containing plasmid were transduced; for a 0.1 ratio, 0.3 μg of C34-conjugated coreceptor, 3.0 μg of unconjugated coreceptor, and 2.7 μg of pcDNA3.1 plasmid were transfected). The transfected cells were incubated over night at 37°C and then plated in duplicate into 24-well plates with each coreceptor in duplicate. The cells were incubated overnight at 37°C and then equal amounts of virus were added to each well. Cells were then incubated for 48 h at 37°C. The level of virus entry was determined by lysing cells with 200 μl of a 0.5% Triton X-100/PBS solution of which 100 μl was then mixed with an equal amount of luciferase substrate (Promega). Luciferase activity was quantified on a Thermo-Labsystems Luminoskan Ascent luminometer.

### Flow cytometry

Cells were aliquoted equally into 13 mm tubes and washed in PBS with 2% FBS. Pelleted cells were then resuspend and stained with particular antibodies on ice for 30 min. CCR5 staining was done with conjugated anti-CCR5 monoclonal antibody 2D7-FITC [fluorescein isothiocyanate] (BD Pharmingen). The anti-CD4 staining was done with mAb #19 [88], and CXCR4 staining was done with mAb 12G5 [88] followed by secondary staining with a FITC-conjugated goat anti-mouse antibody (1:40 dilution; Invitrogen). For the *in vitro* and *in vivo* primary CD4 T cell staining the anti-CD4 was labeled with BV421, clone OKT4, (BioLegend, San Diego, CA) and the anti-p24 Gag antibody, clone KC57, was labeled with RD1 (Beckman Coulter, Brea, CA). C34 staining was done with an anti-C34 mAb, generated at Green Mountain Antibodies (Burlington, VT) by immunizing Balb/c mice with the C34 peptide synthesized at ELIM Biopharmaceuticals (Hayward, CA). Fluorescence-activated cell sorter analysis was performed either on a Becton Dickinson FACS Calibur flow cytometer or a BD LSRII.

### Enfuvirtide-resistant HIV-1

HIV-1 R3A Env was mutagenized using a QuickChange Site-Directed Mutagenesis Kit (Agilent Technologies, Santa Clara, CA). Oligonucleotides primer pairs (refer to **S1 Table** for oligonucleotide sequences) used for the I37A change were I37A_F1/R1; for the V38A were V38A_F1/R1; and for the N43D were N43D_F1/R1. Enfuvirtide was provided by the NIH AIDS Reagent Program Repository and resistance determined with pNL4-3 luc pseudotype virions bearing mutated Envs compared to non-mutated Envs.

### *In vitro* and *in vivo* primary CD4 T Cells

Primary CD4 T cells were enriched by negative selection (UPenn CFAR Immunology Core) and then culture in RPMI supplemented with 300 U rIL-2 / ml and 2mM pen/strep. The cells were stimulated with CD3/CD28 Dynabeads (Invitrogen) for 5 days, de-beaded, and then rested for 3 additional days prior to either *in vitro* HIV-1 challenge or infusion into NSG mice for the *in vivo* challenge.

### HIV-1 isolates

Recombinant NL4-3 virus with the R3A, BaL, and HxB Envs inserted were transfected into 293T cells for 4 h using the standard calcium-phosphate (cal-phos) method. At 48 h post-transfection virus was collected and stored at –80°C. Virus concentrations were quantified via enzyme-linked immunosorbent assay (ELISA) for the viral p24 antigen (Perkin-Elmer). The US1, CMU 02, SF2, JRFL, and MN virus were amplified in PBMC and equal amounts of viral stocks were used in all challenges.

### Viral replication assays

Equivalent amounts of virus were added to all cell lines and at 18 hours post infection excess virus was removed by washing the cells in fresh RPMI 1640 medium supplemented with 10% FBS. Replication was monitored by measuring the viral reverse transcriptase (RT) activity in culture supernatants which were collected at the indicated dates, centrifuged at 45,000 g for 30 min, 4C. The supernatant was aspirated and 100 μl of RT buffer was added to each tube before the samples were stored at -20C until the completion of the experiment.

### Assessment of intracellular cytokines

CD4^+^ T cells were cultured for 12 days after removal of anti-CD3/CD28 coated beads, and then restimulated with PMA/Ionomycin (1.5 ug/mL PMA, 1.0 ug/mL ionomycin; Sigma-Aldrich) or with fresh anti-CD3/CD28 beads (3:1 bead to cell ratio). For PMA/Ionomycin, a 1/1000 dilution of brefeldin-A (BFA, GolgiPlug, Becton Dickinson, cat #555029) was added at the same time, and cells were pulsed for 3 hours before being washed in FACS buffer and fixed in Caltag Fix and Perm buffer A for 15 minutes. Cells restimulated with anti-CD3/CD28 beads were incubated with beads alone for 1 hour, and then with BFA and beads for 4 hours, prior to washing and fixing in buffer A. Cells were washed again in FACS buffer after fixation, and then stained for intracellular cytokines in Caltag Fix and Perm buffer B for 15 minutes. After intracellular staining, cells were washed again in FACS buffer and resuspended in 1X PBS with 2% paraformaldyde, prior to analysis by flow cyotometry.

### Chemotaxis assays

Transendothelial migration of A66-derived CD4+ T cell lines was assessed using a method described previously[89] with some modifications. Briefly, an endothelial cell line, EA.hy926 (ATCC, Manassas, VA), was pre-cultured in 24-well transwell inserts (Coaster) with a 5-μm pore size for 2–4 days in Dulbecco's Modified Eagle's Medium (DMEM) with 10% FBS. Lower wells were then filled with the migration medium (1:1 of RPMI/DMEM, 0.5% BSA, 20 mM HEPES, pH 7.4) in the presence or absence of the indicated amounts of CCL4 or CXCL12. Transwell inserts were washed and filled with 100 μL of A66-derived T cells. The cells were allowed to migrate through the endothelial cell layer into the lower wells at 37°C for 4 h. The migrated cells in the lower well were then collected and counted using a flow-count technique (Coulter). Responses were calculated as the ratio of migrated cells in comparison to total input cells (% input).

### 5-Helix binding assay and quantification of C34-coreceptor surface expression

Surface expression of C34-conjugated coreceptors on A66 cells was quantified by flow fluorimetry using rhodamine-labeled 5-Helix, an engineered partial mimetic of the gp41 6-helix bundle that lacks one of three HR2 domains and, consequently, binds with high affinity and specificity to HR2 [37–39]. A 5-Helix variant containing a C-terminal Cys residue was recombinantly expressed, purified, and conjugated to rhodamine-5-maleimide (Anaspec), as previously [37–39]. Specific binding activity of 5-Helix-rhodamine was assessed through stoichiometric titrations using HR2-peptide C37 (K_D_ = 0.65 pM). Briefly, 5-Helix-rhodamine (estimated concentration of 1 nM) was incubated with varying concentrations of C37 (5 pM—10 nM) for 2 hours at room temperature in Tris-buffered saline containing 100 μg/ml bovine serum albumin. Each solution was individually loaded through the flow cell of a KinExA 3000 flow fluorimeter (Sapidyne Instruments). The flow cell contained azlactone-activated polyacrylamide beads (ThermoFisher) covalently conjugated to C37 peptide. The beads captured free (unbound) 5-Helix-rhodamine, resulting in a change in bead fluorescence (Δ*f*) that was directly proportional to the free 5-Helix concentration in solution. The C37-dependence to Δ*f* was fit using a general bimolecular binding model where the real concentration of 5-Helix-rhodamine was assumed to be unknown (Origin Software, OriginLabs):

$$\Delta f=\Delta f_{\text{min}}+\frac{\Delta f_{\text{max}}-\Delta f_{\text{min}}}{2{\left[5\text{H}\right]}_{0}}\left({\left[5\text{H}\right]}_{0}-{\left[\text{C}37\right]}_{0}-{\text{K}}_{\text{D}}+\sqrt{{\left({\left[5\text{H}\right]}_{0}+{\left[\text{C}37\right]}_{0}+{\text{K}}_{\text{D}}\right)}^{2}-4{\left[5\text{H}\right]}_{0}{\left[\text{C}37\right]}_{0}}\right)$$

Here, Δ*f*_min_ is the minimal fluorescence signal obtained at high C37 concentrations where all 5-Helix-rhodamine is bound; Δ*f*_max_ is the fluorescence signal obtained in the absence of C37; [5H]_0_ and [C37]_0_ are the total concentrations of 5-Helix-rhodamine and C37 used in each incubation; and K_D_ is the equilibrium dissociation constant. The specific binding activity of 5-Helix-rhodamine determined from these experiments (0.61 ± 0.06 nM, compared to the 1 nM estimated concentration) was utilized as the basis for 5-Helix-rhodamine dilutions in cell binding experiments.

To determine expression levels of C34-conjugated coreceptors, A66 cells were first washed extensively in complete RPMI media to remove cellular debris before being visualized with Trypan Blue staining to ensure that the proportion of intact, live cells per sample exceeded 90%. Cells were counted manually with a hemacytometer and subsequently aliquoted (500 μl) at a concentration of 4-8x10^6^ per mL. For titration experiments, C34-conjugated coreceptor-expressing A66 cells were serially diluted (1:2) into an equal number of matching coreceptor-expressing A66 cells so that the total cell concentration for each sample was constant. An equal volume (500 μl) of cell-free media containing 100 pM or 200 pM 5-Helix-rhodamine was added to each cell aliquot and the samples were incubated at room temperature for 2–3 hours. Cells were pelleted by slow speed centrifugation, and supernatants were removed and re-centrifuged at high speeds to remove any additional insoluble material. The amount of 5-Helix-rhodamine remaining in clarified supernatants was quantified by flow fluorimetry as described above. For titration experiments, the dependence of Δ*f* on C34-coreceptor-expressing A66 cell concentration was fit to the bimolecular binding equation above with [5H]_0_ fixed at either 50 or 100 pM and [C37]_0_ replaced with the molar concentration of C34-coreceptors:

$${\left[\text{C}37\right]}_{0}\to {\left[\text{C}34-\text{CoR}\right]}_{0}=\frac{1000\;\text{nc}}{{\text{N}}_{\text{A}}}$$

Here, n is the number of C34-coreceptors per A66 cell (unknown), c is the cell concentration in cells per milliliter, and N_A_ is Avagadro’s number. Δ*f*_min_ was determined from 5-Helix-rhodamine/A66 cell incubations that also included 100 nM C37, while Δ*f*_max_ was obtained from incubations that lacked C34-coreceptor-experessing cells.

### HIV-1 Challenge *in vivo*

NOD.Cg-Prkdc^scid^ Il2rg^tm1Wjl^/SzJ (NSG) mice were engrafted with 10^7^ untransduced or engineered primary human CD4 T cells, and monitored for engraftment of CD45^+^CD4^+^ cells after 3 weeks by TruCount (Becton Dickinson) analysis using 50 μL of blood from each animal. Mice were then normalized based on engraftment into groups of 8–10 animals each per experimental group, and infected with 50 μL each of cell-free US1 and CMU-02 intravenously (50 ng p24 total per animal). Mice were subsequently bled every 10 days prior to necropsy at day 21 or day 28 post-infection, at which point spleens were harvested, processed in 6 mL complete RPMI 1640 (10% FCS) through 70 μm filters, and 50 μL analyzed by TruCount. TruCount analysis was performed using PerCP-Cy5.5 conjugated anti-human CD45 antibody (eBioscience), and BV421-conjugated anti-CD4 antibody (eBioscience). Plasma was saved for viral load analysis when available in sufficient quantities for all animals. Animals were housed at the University of Pennsylvania. Serum viral loads were determined Amplicor HIV-1 Monitor Test (Children's Hospital, Philadelphia), using at least 10 μL of serum per animal.

## Supporting Information

S1 Table Enrichment of C34-conjugated coreceptors following HIV-1 infection. Purified CD4^+^ T cells were transduced with GFP or with lentiviral vectors expressing C34-CCR5 or C34-CXCR4, diluted with untransduced cells to a final concentration of 25%, and inoculated with the indicated HIV-1 isolates. The percentage of viable cells expressing either GFP or the transduced coreceptor, as determined by flow cytometry using an anti-C34 monoclonal antibody, was determined on days 5 and 14 post inoculation. The fold increase in C34-expressing cells from the 25% starting point is shown for C34-CCR5 (yellow) and C34-CXCR4 transduced cells (pink) on day 14. A consistently greater increase expansion of C34-CXCR4 cells is seen (red) compared to C34-CCR5 cells (yellow). The average of 2 experiments with different donor cells for each virus is shown.
(DOCX)

S2 Table Sequences of oligonucleotides used to generate C34-conjugated constructs of CXCR4, CCR5 and CD4, and enfuvirtide-resistant HIV-1. Oligonucleotide primers are shown that were used to generate constructs of CXCR4, CCR5 and CD4 containing the C34 peptide from the HIV-1 gp41 HR2 domain conjugated to their amino termini. Primer pairs used to generate enfuvirtide-resistant isolates of HIV-1/R3A are also shown.
(DOCX)

S1 Fig Diagram showing the design of C34-containing CXCR4 and CCR5 coreceptors. Top panel shows HIV-1 gp41 with regions highlighted: FP (fusion peptide), heptad repeat 1 (HR1), heptad repeat 2 (HR2), MSD (membrane spanning domain), and the cytoplasmic domain (CD). Amino acid locations relative to the start of the *env* open reading frame are indicated (HXB2 numbering). The 34 amino acid peptide (C34) from within HR2 is indicated in with an N-terminal Ala, and a C-terminal Leu-Lys introduced as linkers. The two lower panels show the insertion sites for the C34 peptide (plus the linker) into the amino termini of CXCR4 and CCR5. See Methods for construction strategy.
(TIF)

S2 Fig Chemokine-induced transendothelial migration of CD4+ T cell lines mediated by C34-conjugated coreceptors. Migration of A66 stably expressing the indicated coreceptors, unconjugated or conjugated to C34 peptide, was assessed on 5-μm transwell membranes in which the indicated amounts of chemokines CXCL12 **(A)** or CCL4 **(B)** were added to the lower wells. Shown are mean ± s.d. of cells entering the lower well. A representative experiment of two (**A**) or three (**B**) independent experiments is shown.
(TIF)

S3 Fig Inhibition of diverse HIV-1 isolates by C34-conjugated coreceptors. Infection of A66 cells stably expressing the indicated coreceptors is shown following inoculation by R5-tropic HIV-1 isolates YU2 **(A)** and JRFL **(B)** and dual-tropic R3A **(C)**. Infection was monitored over time by reverse transcriptase (RT) activity.
(TIF)

S4 Fig 5-Helix binding and quantifying surface expression levels of C34-conjugated coreceptors on A66 cells. **(A and B)** A66 cells were incubated with rhodamine-labeled 5-Helix, an engineered protein that binds HR2-derived peptides with high affinity [37–39]. Receptor-bound 5-Helix was removed by centrifugation, and the amount of 5-Helix remaining in supernatants assessed by flow fluorimetry (KinExA 3000, Sapidyne Instruments). Each sample was loaded through a flow cell containing HR2-peptide-coated beads that bind 5-Helix. The change in bead fluorescence (Δ*f*) measured before sample load and after washout was directly proportional to the amount of rhodamine-labeled 5-Helix in the supernatant. **(C)** Fluorescence traces obtained from incubations of 5-Helix (100 pM) with mixtures of C34-CXCR4-expressing and CXCR4-expressing A66 cells. A background signal (Δ*f*_min_) was obtained by including enough HR2-peptide C37 (100 nM, K_D_ = 0.65 pM) to bind all 5-Helix and block its interaction with beads in the flow cell (dashed trace). **(D)** Fluorescence signals (Δ*f*) obtained from incubations of 5-Helix (100 pM) with culture media only, parental A66 cells, A66 cells expressing CXCR4 with or without 100 nM of C37 peptide, and A66 cells expressing C34-conjugated CXCR4. The comparable Δ*f* values for culture media only, parental A66 and CXCR4-expressing A66 cells indicates that 5-Helix has minimal nonspecific interactions with A66 cells or CXCR4. The reduction in Δ*f* value for the C34-CXCR4-expressing A66 cells indicates a specific interaction between 5-Helix and the HR2-conjugated coreceptor. **(E)** Fluorescence signals (Δ*f*) measured for incubations of 5-Helix (100 pM) with increasing concentrations of C34-CXCR4-expressing A66 cells. Total cell concentration in each incubation was maintained at 2x10^6^ using CXCR4-expressing A66 cells. The open square represents a measurement of Δ*f*_min_ from an incubation that included 100 mM C37 peptide (see Methods). **(F and G)** Data are shown as in Panels D and E, except that C34-CCR5-expressing and CCR5-expressing A66 cells were interrogated. The data and error bars in Panels D through G represent the mean and range-of-mean of duplicate measurements of a single experiment. Δ*f* values in Panels E and G were fit (solid lines) to a general bimolecular binding model (see Methods) to determine the number of C34-conjugated coreceptors per A66 cell and approximate K_D_ values. Experiments were repeated at least three times for both C34-CXCR4-expressing and C34-CCR5-expressing A66 cells, yielding average receptor densities of 55,000 ±13,000 and 52,000 ± 11,000 per cell, respectively, and K_D_ values of approximately 4 ± 1 and 13 ± 6 pM, respectively.
(TIF)

S5 Fig Surface expression of C34-conjugated coreceptors on A66 cells. A66 cells (i.e. SupT1 cells, ablated for CXCR4 expression through zinc finger nuclease treatment [36]) were transduced to express either wild type or C34-conjugated coreceptors, and surface expression assessed with mAbs to CCR5 (3A9) or CXCR4 (12G5), as well as a mAb to the C34 peptide. Relative to untransduced A66 cells, transduced cells show high and specific levels of expression of conjugated and C34-conjugated receptors.
(TIF)

S6 Fig SIVmac239 infection is not inhibited by C34-CXCR4 when co-expressed with CCR5. A66 cells stably expressing CCR5 alone or CCR5 with C34-conjugagted CXCR4 were incubated with SIVmac239 (50 ng of p27-Gag) overnight. Cells were washed to remove input virus, and reverse transcriptase activity (RT) in culture supernatant determined. SIVmac239 replication occurred to high levels on both cell types. Parental A66 cells, lacking CCR5 or CXCR4 and used as a control, remained uninfected. When inoculations of these same cells were performed with an R5-tropic isolate of HIV-1 (BaL), infection occurred on CCR5-expressing cells but was completely inhibited on cells co-expressing CCR5 and C34-conjugated CXCR4 (**Fig 2B**).
(TIF)

S7 Fig Intracellular cytokine expression in activated CD4 T cells expressing C34-conjugated coreceptors. CD4 T cells from the normal donors that either untransduced (T cell) or transduced with GFP or the indicated C34-conjugated constructs were stimulated as indicated (PHA/ionomycin or CD3/CD28 Dynabeads) or not stimulated and expression of intracellular cytokines assessed. Shown are cytograms for CCL4 (MIP-1β) and interferon-γ (**Panel A**) and TNFα and IL-2 (**Panel B).** Levels of cytokines were comparable between all groups of cells.
(TIF)

S8 Fig Kinetics of C34-coreceptor inhibition of HIV-1 in primary CD4 T cells. For primary CD4 T cells either untransduced or transduced with the indicated C34-conjugated constructs or a GFP control, p24-Gag expression was assessed over time by flow cytometry following inoculation with HIV-1 isolate JRFL. Results are shown in cultures containing only transduced cells (**A**) or mixtures containing a 1:3 ratio of transduced to untransduced cells (**B**), as described in **Fig 5**.
(TIF)

S9 Fig Primary CD4 T cells expressing C34-conjugated coreceptors are protected from infection by dual-tropic HIV-1. Primary human CD4 T cells were transduced with the indicated C34-conjugated constructs or GFP control, inoculated with dual-tropic HIV-1 R3A, and monitored by flow cytometry with an anti-C34 peptide antibody and intracellular p24-Gag expression. **(A)** Cells inoculated with R3A show stable expression of C34-constructs at days 5 and 14 with marked inhibition of p24-Gag expression in C34-CCR5 and C34-CXCR4 transduced cultures relative to untransduced, GFP- or C34-CD4 transduced cells. **(B)** Cells transduced with C34-CCR5, C34-CXCR4, C34-CD4 or GFP were added at a 1:3 ratio to untransduced cells as in **Fig 5**, and inoculated with R3A. Expansion of cells expressing C34-CCR5 or C34-CXCR4 is shown over time.
(TIF)
