# Supplementary material for: Potent and Broad Inhibition of HIV-1 by a Peptide from the gp41 Heptad Repeat-2 Domain Conjugated to the CXCR4 Amino Terminus
Source: PLoS Pathog. 2016 Nov 17;12(11):e1005983. doi: 10.1371/journal.ppat.1005983 (PMC5113989; doi:10.1371/journal.ppat.1005983)
Supplement: S1 Table — Purified CD4+ T cells were transduced with GFP or with lentiviral vectors expressing C34-CCR5 or C34-CXCR4, diluted with untransduced cells to a final concentration of 25%, and inoculated with the indicated HIV-1 isolates. The percentage of viable cells expressing either GFP or the transduced coreceptor, as determined by flow cytometry using an anti-C34 monoclonal antibody, was determined on days 5 and 14 post inoculation. The fold increase in C34-expressing cells from the 25% starting point is shown for C34-CCR5 (yellow) and C34-CXCR4 transduced cells (pink) on day 14. A consistently greater increase expansion of C34-CXCR4 cells is seen (red) compared to C34-CCR5 cells (yellow). The average of 2 experiments with different donor cells for each virus is shown. (DOCX) [file ppat.1005983.s001.docx]

**S1 Table. Enrichment of C34-conjugated coreceptors following HIV-1 infection**

|  | **GFP** | | **C34-CCR5** | | | **C34-CXCR4** | | |
| --- | --- | --- | --- | --- | --- | --- | --- | --- |
| **Virus** | Day 5 | Day 14 | Day 5 | Day 14 | fold increase @ Day14 | Day 5 | Day 14 | fold increase @ Day14 |
| No Virus | 23.2 | 18.2 | 22.5 | 20.7 | **0.83** | 25.3 | 30.4 | **1.22** |
| JRFL | 22.3 | 12.0 | 26.0 | 41.8 | **1.67** | 29.1 | 61.4 | **2.46** |
| BaL | 22.8 | 20.4 | 26.7 | 37.0 | **1.48** | 31.3 | 48.8 | **1.95** |
| US1 | 22.9 | 12.8 | 25.1 | 41.0 | **1.64** | 27.2 | 57.6 | **2.30** |
| CMU-02 | 23.0 | 14.8 | 23.0 | 47.7 | **1.91** | 25.4 | 63.3 | **2.53** |
| MN | 23.3 | 15.8 | 23.4 | 53.6 | **2.14** | 26.5 | 72.6 | **2.90** |
| SF2 | 22.9 | 18.2 | 23.7 | 46.5 | **1.86** | 26.3 | 66.1 | **2.64** |
| R3A | 23.2 | 18.8 | 35.8 | 49.2 | **1.97** | 39.9 | 70.8 | **2.83** |
