# Supplementary material for: Potent and Broad Inhibition of HIV-1 by a Peptide from the gp41 Heptad Repeat-2 Domain Conjugated to the CXCR4 Amino Terminus
Source: PLoS Pathog. 2016 Nov 17;12(11):e1005983. doi: 10.1371/journal.ppat.1005983 (PMC5113989; doi:10.1371/journal.ppat.1005983)
Supplement: S2 Table — Oligonucleotide primers are shown that were used to generate constructs of CXCR4, CCR5 and CD4 containing the C34 peptide from the HIV-1 gp41 HR2 domain conjugated to their amino termini. Primer pairs used to generate enfuvirtide-resistant isolates of HIV-1/R3A are also shown. (DOCX) [file ppat.1005983.s002.docx]

**S2 Table. Sequences of oligonucleotides used to generate C34-conjugated constructs of CXCR4, CCR5 and CD4, and enfuvirtide-resistant HIV-1**

| **Oligo name** | **Oligo sequence** |
| --- | --- |
| C34-left-F1 | 5’-CTAGCACCATGTGGATGGAGTGGGACAGAGAGATCAACAACTACACCAGCCTGAT-3’ |
| C34-left-R1 | 5'-GGATGGTCTCTGTGGATCAGGCTGGTGTAGTTGTTGATCTCTCTGTCCCACTCCATCCACATGGTGCTAGCAGGA-3' |
| C34-right-F1 | 5'-CTCTGGTCTCTCCACAGCCTGATCGAGGAGAGCCAGAACCAGCAGGAGAAGAACGAGCAGGAGCTGCTGCTTAAGATCCTG-3' |
| C34-right-R1 | 5’-TTAAGCAGCAGCTCCTGCTCGTTCTTCTCCTGCTGGTTCTGGCTCTCCTCGATCAGGCT-3' |
| SC34mut4-left-F1 | 5’-CTAGCACCATGTGGATGGAGTTCGACAGAGAGCTGAACAACTACACCAGCCTGAT-3’ |
| SC34mut4-left-R1 | 5’-GTGGATCAGGCTGGTGTAGTTGTTCAGCTCTCTGTCGAACTCCATCCACATGGTG-3’ |
| SC34mut4-right-F1 | 5’-CCACAGCCTGATCGAGGAGAGCCAGAACGAGCAGGAGAAGGACGAGCAGGAGCTGCTGC-3’ |
| SC34mut4-right-R1 | 5’-TTAAGCAGCAGCTCCTGCTCGTCCTTCTCCTGCTCGTTCTGGCTCTCCTCGATCAGGCT-3’ |
| SC34mut8-left-F1 | 5’-CTAGCACCATGTGGATGGAGTTCGACAGAGAGCTGAACAACTTCACCAGCCTGCT-3’ |
| SC34mut8-left-R1 | 5’-GTGCAGCAGGCTGGTGAAGTTGTTCAGCTCTCTGTCGAACTCCATCCACATGGTG-3’ |
| SC34mut8-right-F1 | 5’-GCACAGCATCATCGAGGAGGCCCAGAACGAGCAGGAGAAGGACGAGCAGGAGCTGCTGC-3’ |
| SC34mut8-right-R1 | 5’-TTAAGCAGCAGCTCCTGCTCGTCCTTCTCCTGCTCGTTCTGGGCCTCCTCGATGATGCT-3’ |
| R5 cDNA_F1 | 5'-GTGGTCTCTTAAGGATTATCAAGTGTCAAGTCCAATC-3' |
| R5 cDNA_R1 | 5'-CAGTACACCTCGAGTCACAAGCCCACAGATATTTCCTGC-3' |
| CD4sig_F1 | 5'-GAAGAGACTAGTACCATGAACCGGGGAGTCCCTTTTAG-3' |
| CD4sig_R1 | 5'-GTGGTTGGTCTCCATCCACAGCACCACTTTCTTTCCCTG-3' |
| C34_F1 | 5'-GTGGTTGGTCTCTGGATGGAGTGGGACAGAGAG-3 |
| C34_R1 | 5'-GATGGTGAATTCCAGCAGCTCCTGCTCGTTCTTC-3' |
| CD4_cDNA_F1 | 5'-CTACCAGAATTCAAGAAAGTGGTGCTGGGCAA-3' |
| CD4_cDNA_R1 | 5'-GACAAGAGATCTTCAAATGGGGCTACATGTCTTC-3' |
| I37A_F1 | 5’-GACTATTATTGTCTGGTAAAGTGCAACAGCAG-3’ |
| I37A_R1 | 5’-CTGCTGTTGCACTTTACCAGACAATAATAGTC-3’ |
| V38A_F1 | 5’-GTCTGGTATAGCGCAACAGCAGAACAATCTG-3’ |
| V38A_R1 | 5’-CAGATTGTTCTGCTGTTGCGCTATACCAGA-3’ |
| N43D_F1 | 5’-GCAACAGCAGAACGATCTGCTGAGGG-3’ |
| N43D_R1 | 5’-CCCTCAGCAGATCGTTCTGCTGTTGC-3’ |
