# Supplementary figures and images for: Potent and Broad Inhibition of HIV-1 by a Peptide from the gp41 Heptad Repeat-2 Domain Conjugated to the CXCR4 Amino Terminus
Source: PLoS Pathog. 2016 Nov 17;12(11):e1005983. doi: 10.1371/journal.ppat.1005983 (PMC5113989; doi:10.1371/journal.ppat.1005983)

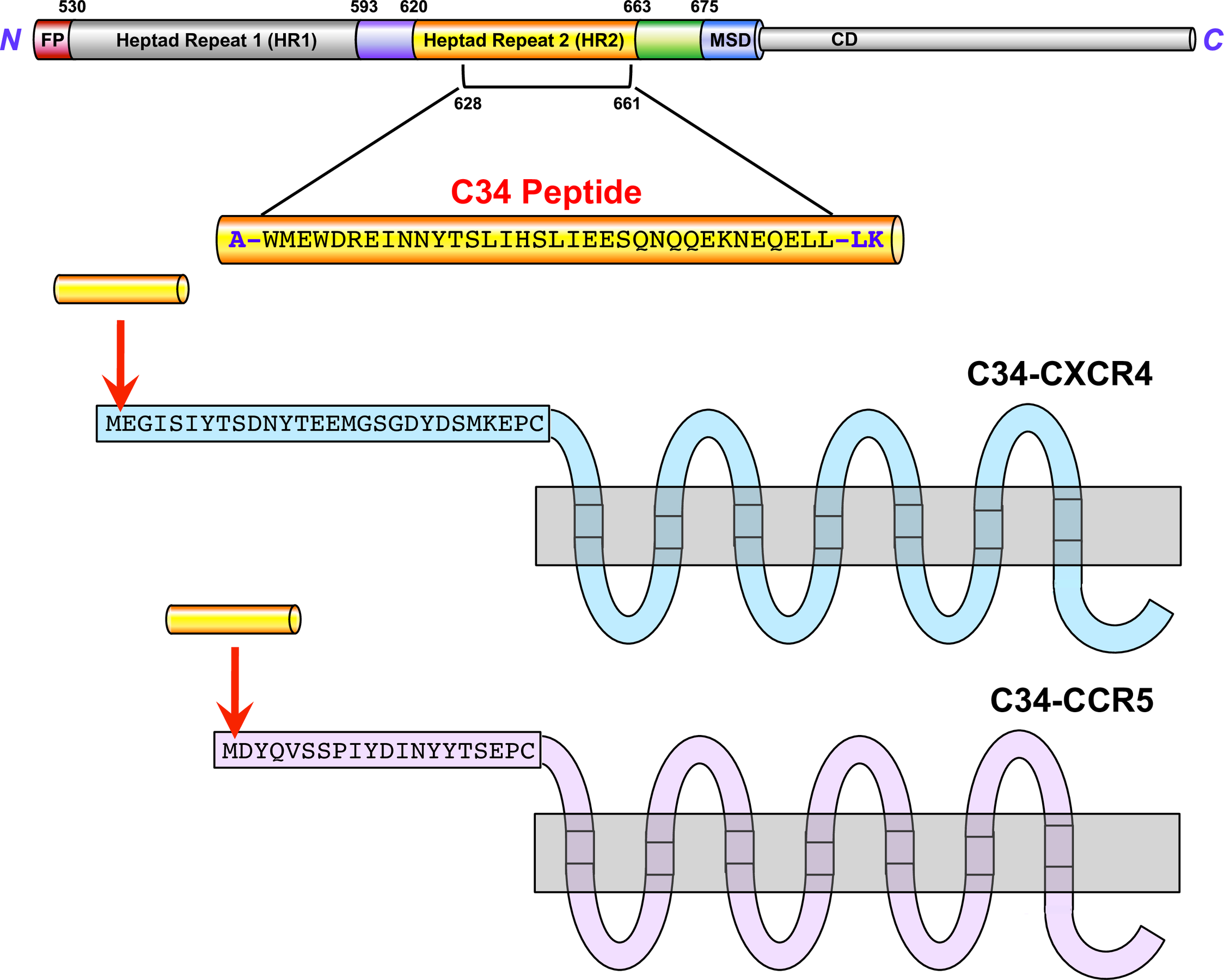

Supplement: S1 Fig — Top panel shows HIV-1 gp41 with regions highlighted: FP (fusion peptide), heptad repeat 1 (HR1), heptad repeat 2 (HR2), MSD (membrane spanning domain), and the cytoplasmic domain (CD). Amino acid locations relative to the start of the env open reading frame are indicated (HXB2 numbering). The 34 amino acid peptide (C34) from within HR2 is indicated in with an N-terminal Ala, and a C-terminal Leu-Lys introduced as linkers. The two lower panels show the insertion sites for the C34 peptide (plus the linker) into the amino termini of CXCR4 and CCR5. See Methods for construction strategy. (TIF) [file ppat.1005983.s003.tif]

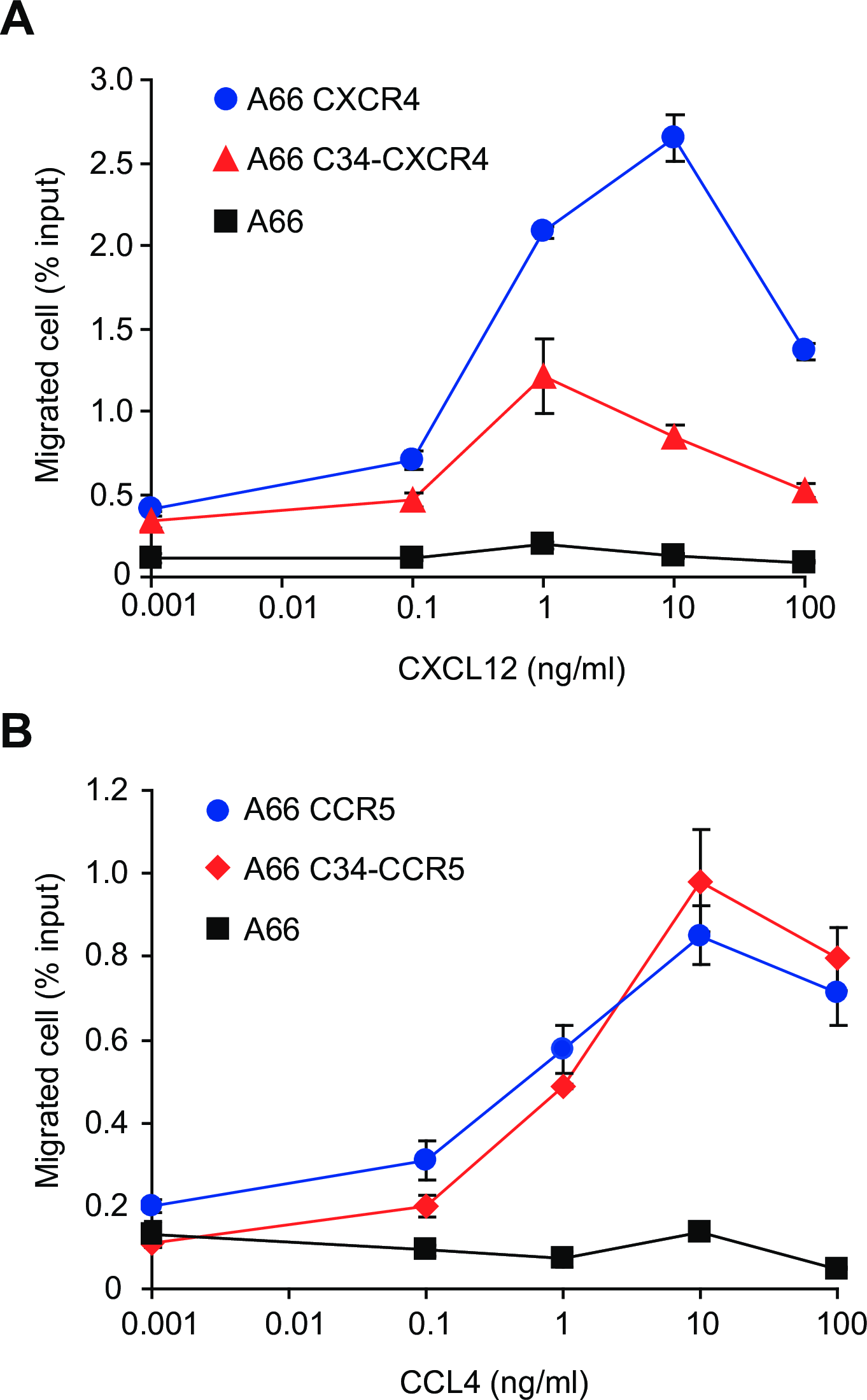

Supplement: S2 Fig — Migration of A66 stably expressing the indicated coreceptors, unconjugated or conjugated to C34 peptide, was assessed on 5-μm transwell membranes in which the indicated amounts of chemokines CXCL12 (A) or CCL4 (B) were added to the lower wells. Shown are mean ± s.d. of cells entering the lower well. A representative experiment of two (A) or three (B) independent experiments is shown. (TIF) [file ppat.1005983.s004.tif]

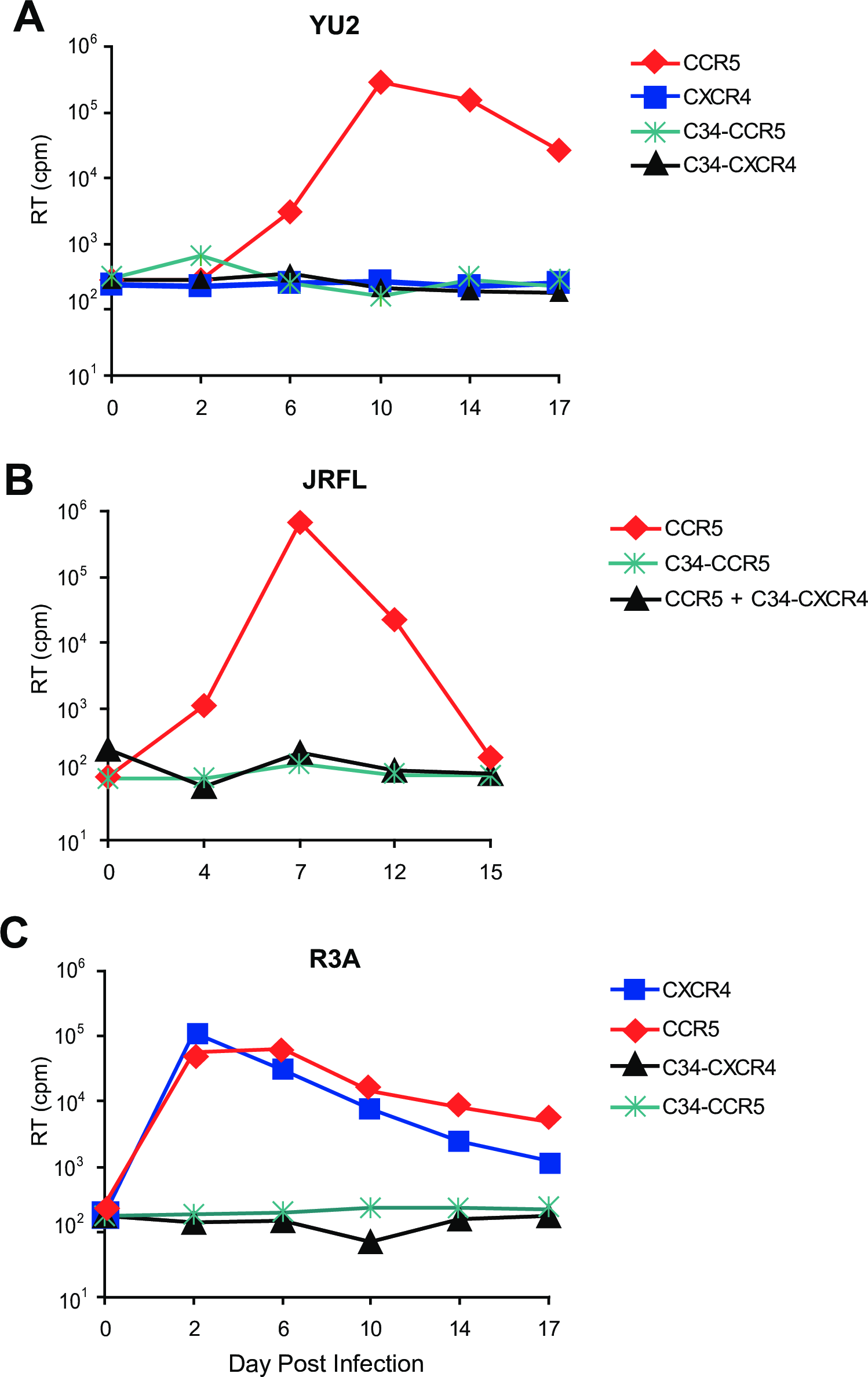

Supplement: S3 Fig — Infection of A66 cells stably expressing the indicated coreceptors is shown following inoculation by R5-tropic HIV-1 isolates YU2 (A) and JRFL (B) and dual-tropic R3A (C). Infection was monitored over time by reverse transcriptase (RT) activity. (TIF) [file ppat.1005983.s005.tif]

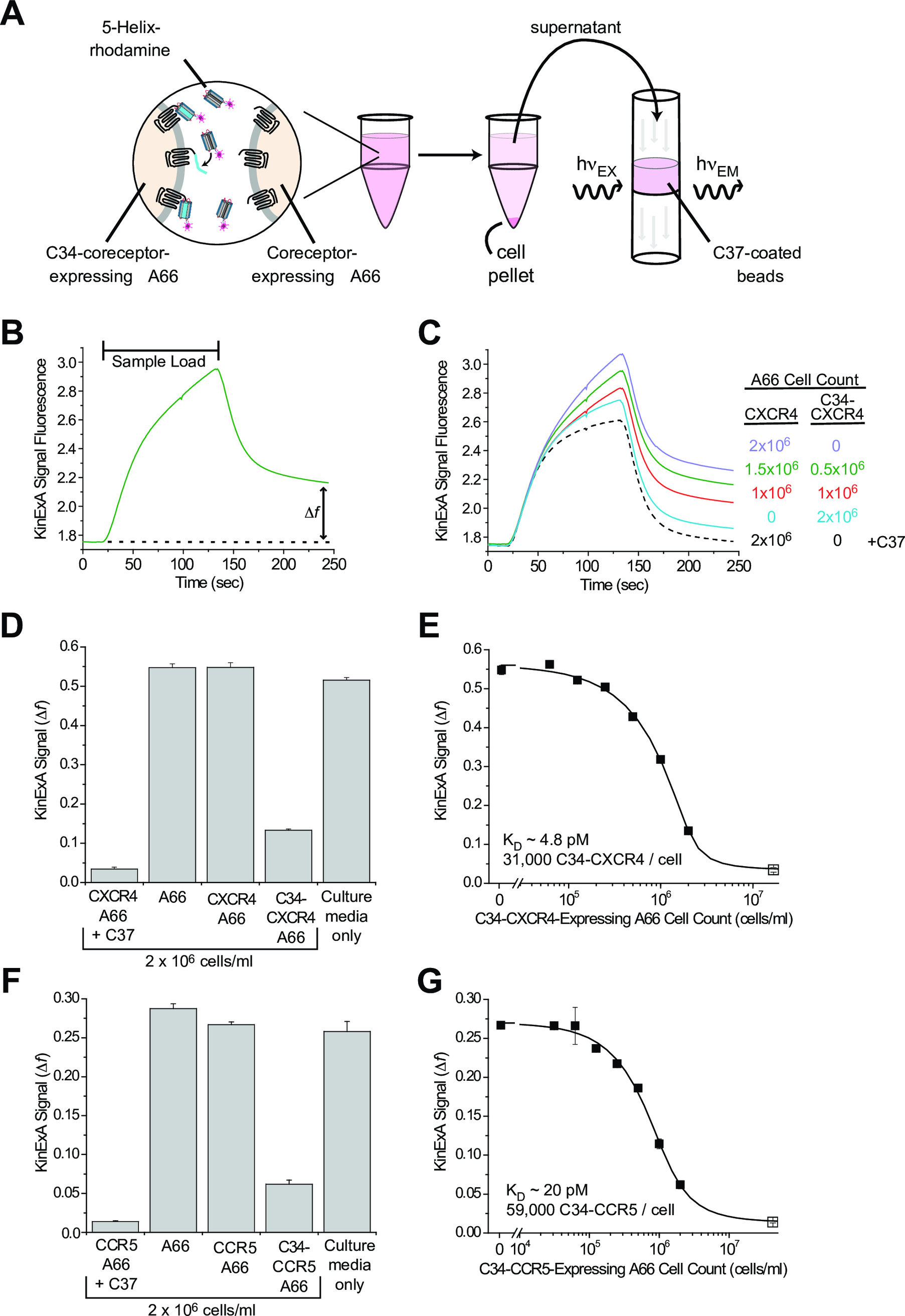

Supplement: S4 Fig — (A and B) A66 cells were incubated with rhodamine-labeled 5-Helix, an engineered protein that binds HR2-derived peptides with high affinity [37–39]. Receptor-bound 5-Helix was removed by centrifugation, and the amount of 5-Helix remaining in supernatants assessed by flow fluorimetry (KinExA 3000, Sapidyne Instruments). Each sample was loaded through a flow cell containing HR2-peptide-coated beads that bind 5-Helix. The change in bead fluorescence (Δf) measured before sample load and after washout was directly proportional to the amount of rhodamine-labeled 5-Helix in the supernatant. (C) Fluorescence traces obtained from incubations of 5-Helix (100 pM) with mixtures of C34-CXCR4-expressing and CXCR4-expressing A66 cells. A background signal (Δf min) was obtained by including enough HR2-peptide C37 (100 nM, KD = 0.65 pM) to bind all 5-Helix and block its interaction with beads in the flow cell (dashed trace). (D) Fluorescence signals (Δf) obtained from incubations of 5-Helix (100 pM) with culture media only, parental A66 cells, A66 cells expressing CXCR4 with or without 100 nM of C37 peptide, and A66 cells expressing C34-conjugated CXCR4. The comparable Δf values for culture media only, parental A66 and CXCR4-expressing A66 cells indicates that 5-Helix has minimal nonspecific interactions with A66 cells or CXCR4. The reduction in Δf value for the C34-CXCR4-expressing A66 cells indicates a specific interaction between 5-Helix and the HR2-conjugated coreceptor. (E) Fluorescence signals (Δf) measured for incubations of 5-Helix (100 pM) with increasing concentrations of C34-CXCR4-expressing A66 cells. Total cell concentration in each incubation was maintained at 2x10^6^ using CXCR4-expressing A66 cells. The open square represents a measurement of Δf min from an incubation that included 100 mM C37 peptide (see Methods). (F and G) Data are shown as in Panels D and E, except that C34-CCR5-expressing and CCR5-expressing A66 cells were interrogated. The data and error bars in P [file ppat.1005983.s006.tif]

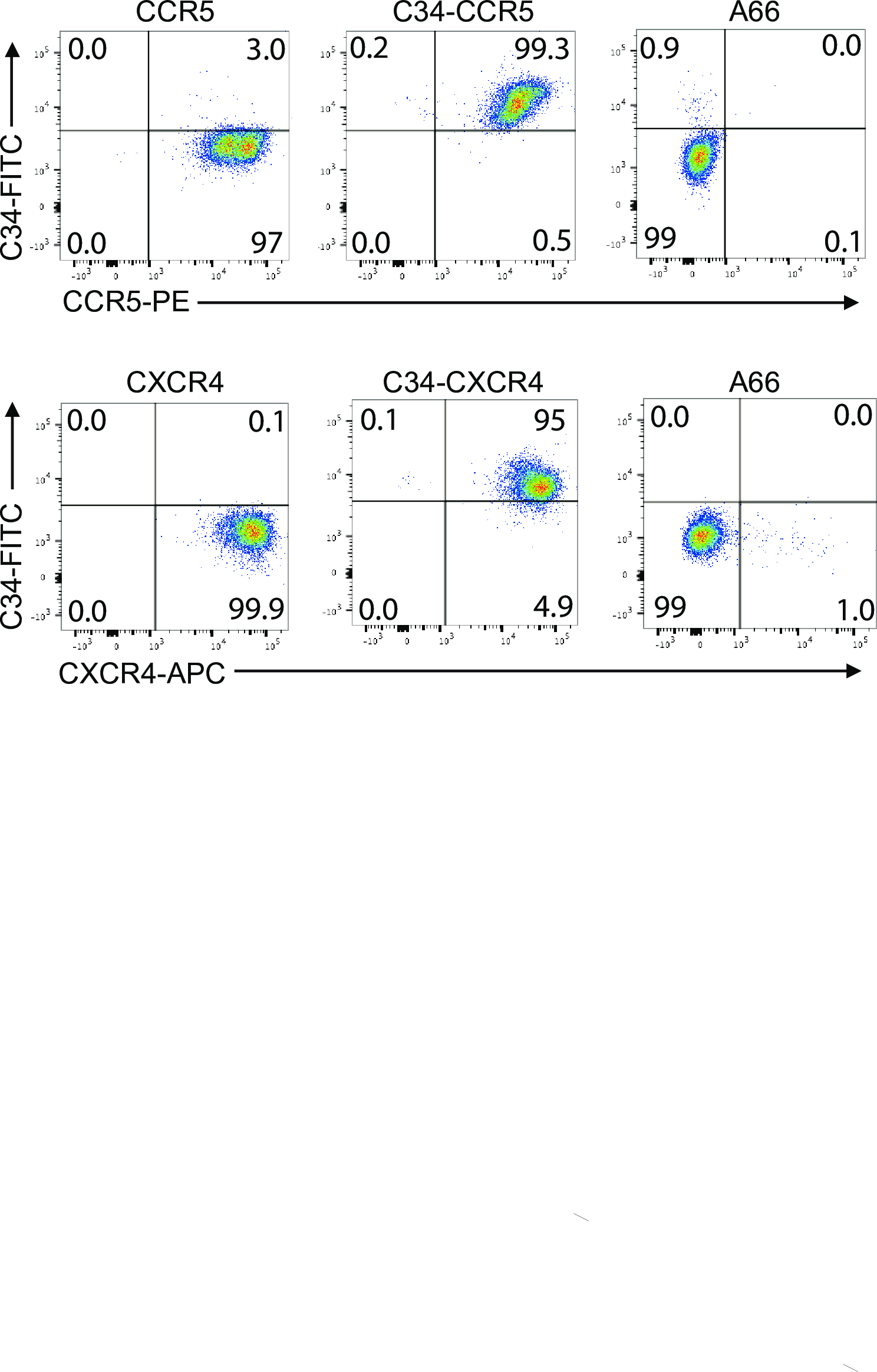

Supplement: S5 Fig — A66 cells (i.e. SupT1 cells, ablated for CXCR4 expression through zinc finger nuclease treatment [36]) were transduced to express either wild type or C34-conjugated coreceptors, and surface expression assessed with mAbs to CCR5 (3A9) or CXCR4 (12G5), as well as a mAb to the C34 peptide. Relative to untransduced A66 cells, transduced cells show high and specific levels of expression of conjugated and C34-conjugated receptors. (TIF) [file ppat.1005983.s007.tif]

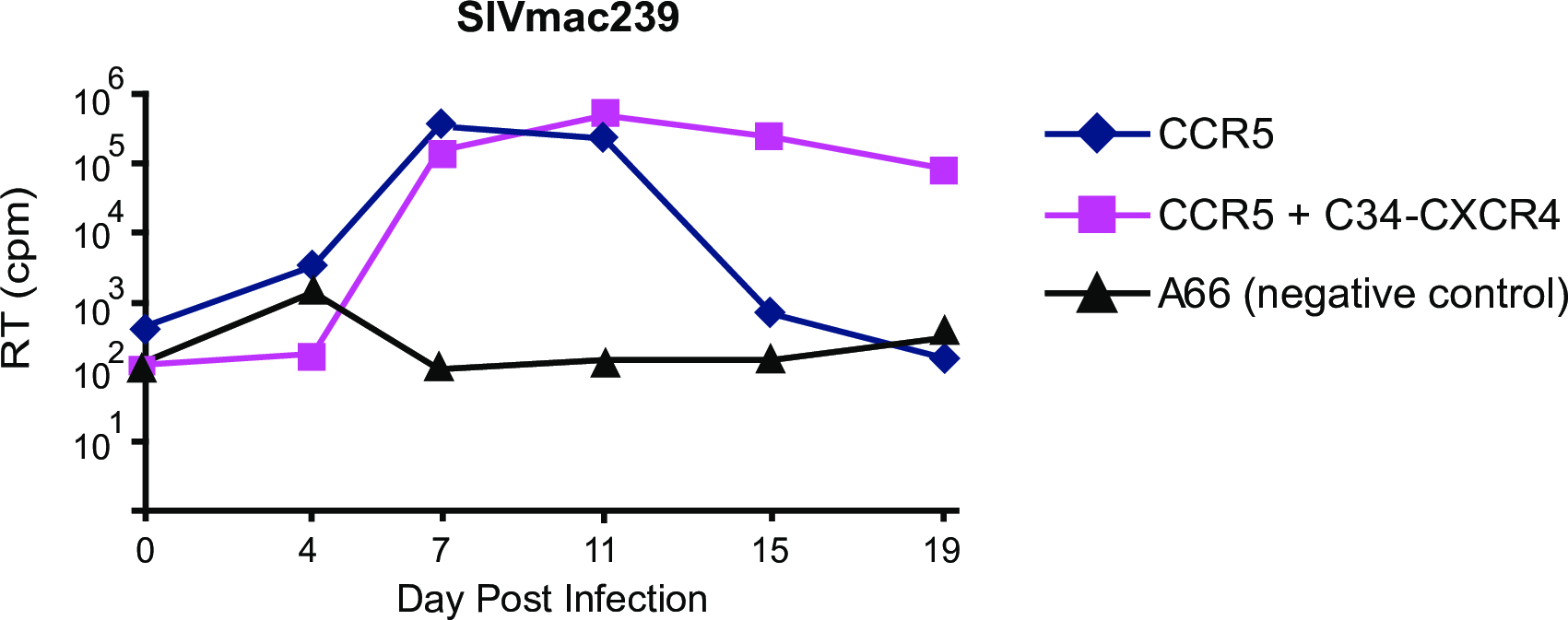

Supplement: S6 Fig — A66 cells stably expressing CCR5 alone or CCR5 with C34-conjugagted CXCR4 were incubated with SIVmac239 (50 ng of p27-Gag) overnight. Cells were washed to remove input virus, and reverse transcriptase activity (RT) in culture supernatant determined. SIVmac239 replication occurred to high levels on both cell types. Parental A66 cells, lacking CCR5 or CXCR4 and used as a control, remained uninfected. When inoculations of these same cells were performed with an R5-tropic isolate of HIV-1 (BaL), infection occurred on CCR5-expressing cells but was completely inhibited on cells co-expressing CCR5 and C34-conjugated CXCR4 (Fig 2B). (TIF) [file ppat.1005983.s008.tif]

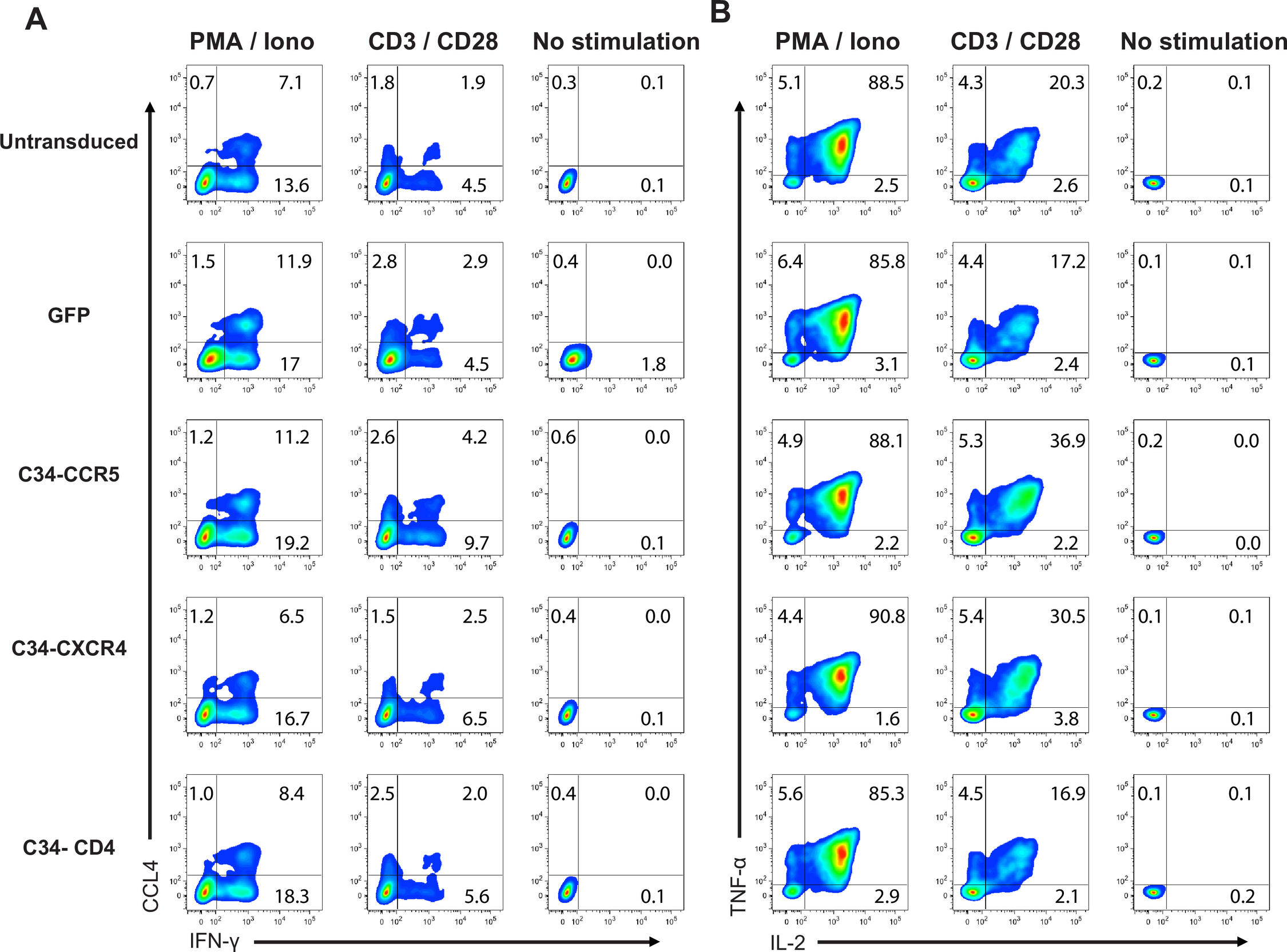

Supplement: S7 Fig — CD4 T cells from the normal donors that either untransduced (T cell) or transduced with GFP or the indicated C34-conjugated constructs were stimulated as indicated (PHA/ionomycin or CD3/CD28 Dynabeads) or not stimulated and expression of intracellular cytokines assessed. Shown are cytograms for CCL4 (MIP-1β) and interferon-γ (Panel A) and TNFα and IL-2 (Panel B). Levels of cytokines were comparable between all groups of cells. (TIF) [file ppat.1005983.s009.tif]

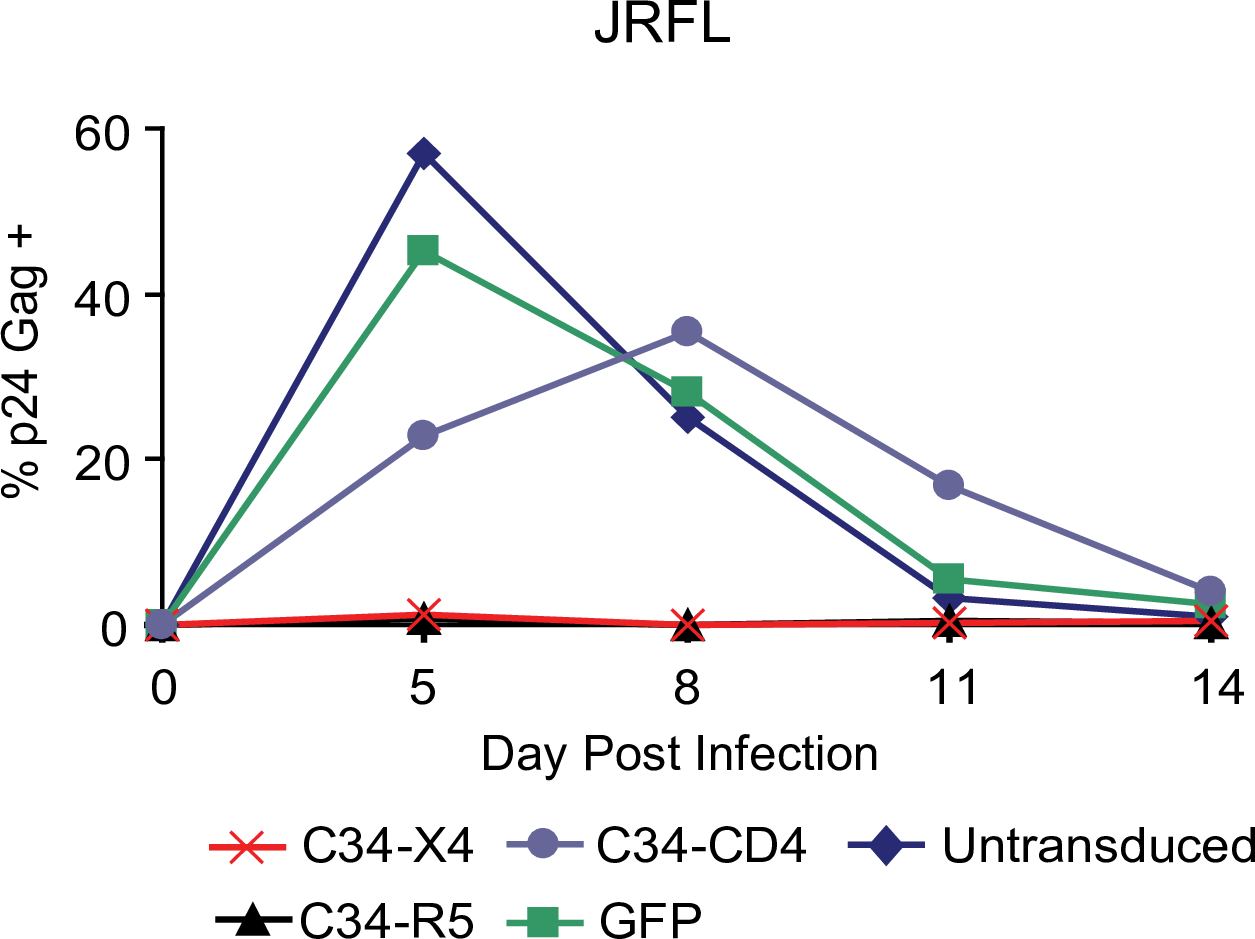

Supplement: S8 Fig — For primary CD4 T cells either untransduced or transduced with the indicated C34-conjugated constructs or a GFP control, p24-Gag expression was assessed over time by flow cytometry following inoculation with HIV-1 isolate JRFL. Results are shown in cultures containing only transduced cells (A) or mixtures containing a 1:3 ratio of transduced to untransduced cells (B), as described in Fig 5. (TIF) [file ppat.1005983.s010.tif]

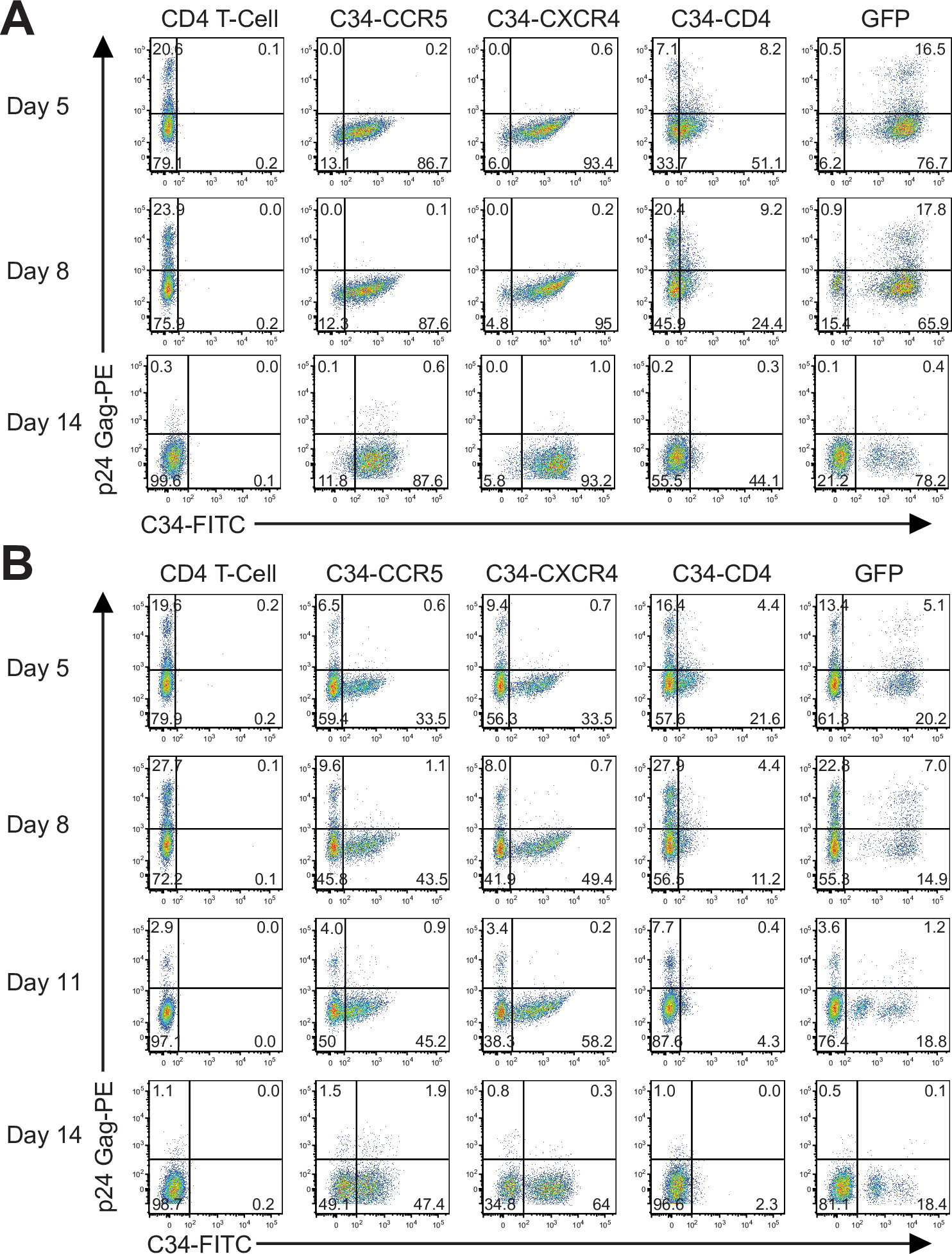

Supplement: S9 Fig — Primary human CD4 T cells were transduced with the indicated C34-conjugated constructs or GFP control, inoculated with dual-tropic HIV-1 R3A, and monitored by flow cytometry with an anti-C34 peptide antibody and intracellular p24-Gag expression. (A) Cells inoculated with R3A show stable expression of C34-constructs at days 5 and 14 with marked inhibition of p24-Gag expression in C34-CCR5 and C34-CXCR4 transduced cultures relative to untransduced, GFP- or C34-CD4 transduced cells. (B) Cells transduced with C34-CCR5, C34-CXCR4, C34-CD4 or GFP were added at a 1:3 ratio to untransduced cells as in Fig 5, and inoculated with R3A. Expansion of cells expressing C34-CCR5 or C34-CXCR4 is shown over time. (TIF) [file ppat.1005983.s011.tif]
